# Phenomics based prediction of plant biomass and leaf area in wheat using machine learning approaches

**DOI:** 10.3389/fpls.2023.1214801

**Published:** 2023-06-28

**Authors:** Biswabiplab Singh, Sudhir Kumar, Allimuthu Elangovan, Devendra Vasht, Sunny Arya, Nguyen Trung Duc, Pooja Swami, Godawari Shivaji Pawar, Dhandapani Raju, Hari Krishna, Lekshmy Sathee, Monika Dalal, Rabi Narayan Sahoo, Viswanathan Chinnusamy

**Affiliations:** ^1^ Division of Plant Physiology and Nanaji Deshmukh Plant Phenomics Centre (NDPPC), Indian Council of Agricultural Research (ICAR)-Indian Agricultural Research Institute, New Delhi, India; ^2^ Vietnam National University of Agriculture, Hanoi, Vietnam; ^3^ Division of Agricultural Botany, Vasantrao Naik Marathwada Krishi Vidyapeeth, Parbhani, India; ^4^ Division of Genetics, ICAR-Indian Agricultural Research Institute, New Delhi, India; ^5^ ICAR-National Institute for Plant Biotechnology, New Delhi, India; ^6^ Division of Agricultural Physics, ICAR-Indian Agricultural Research Institute, New Delhi, India

**Keywords:** high-throughput phenotyping (HTP), RGB image, NIR image, machine learning, i-traits, wheat, shoot area

## Abstract

**Introduction:**

Phenomics has emerged as important tool to bridge the genotype-phenotype gap. To dissect complex traits such as highly dynamic plant growth, and quantification of its component traits over a different growth phase of plant will immensely help dissect genetic basis of biomass production. Based on RGB images, models have been developed to predict biomass recently. However, it is very challenging to find a model performing stable across experiments. In this study, we recorded RGB and NIR images of wheat germplasm and Recombinant Inbred Lines (RILs) of Raj3765xHD2329, and examined the use of multimodal images from RGB, NIR sensors and machine learning models to predict biomass and leaf area non-invasively.

**Results:**

The image-based traits (i-Traits) containing geometric features, RGB based indices, RGB colour classes and NIR features were categorized into architectural traits and physiological traits. Total 77 i-Traits were selected for prediction of biomass and leaf area consisting of 35 architectural and 42 physiological traits. We have shown that different biomass related traits such as fresh weight, dry weight and shoot area can be predicted accurately from RGB and NIR images using 16 machine learning models. We applied the models on two consecutive years of experiments and found that measurement accuracies were similar suggesting the generalized nature of models. Results showed that all biomass-related traits could be estimated with about 90% accuracy but the performance of model BLASSO was relatively stable and high in all the traits and experiments. The R^2^ of BLASSO for fresh weight prediction was 0.96 (both year experiments), for dry weight prediction was 0.90 (Experiment 1) and 0.93 (Experiment 2) and for shoot area prediction 0.96 (Experiment 1) and 0.93 (Experiment 2). Also, the RMSRE of BLASSO for fresh weight prediction was 0.53 (Experiment 1) and 0.24 (Experiment 2), for dry weight prediction was 0.85 (Experiment 1) and 0.25 (Experiment 2) and for shoot area prediction 0.59 (Experiment 1) and 0.53 (Experiment 2).

**Discussion:**

Based on the quantification power analysis of i-Traits, the determinants of biomass accumulation were found which contains both architectural and physiological traits. The best predictor i-Trait for fresh weight and dry weight prediction was Area_SV and for shoot area prediction was projected shoot area. These results will be helpful for identification and genetic basis dissection of major determinants of biomass accumulation and also non-invasive high throughput estimation of plant growth during different phenological stages can identify hitherto uncovered genes for biomass production and its deployment in crop improvement for breaking the yield plateau.

## Introduction

Wheat (*Triticum aestivum* L.) is one of the most important cereal crops in the world since the beginning of agriculture, feeding nearly 40% of the world’s population (Giraldo et al., 2019). It is grown in about 217 million hectares across the globe, with an annual production of about around 731 million tonnes (Ramadas et al., 2020). Wheat crop production needs to be increased at least by 60%, to feed the 10-billion people by 2050 (Misra et al., 2020). India is the world’s second-largest wheat producer and a major exporter of wheat. Hence wheat crop is given emphasis in crop improvement programs in India as well as in different countries for breeding better wheat varieties with enhanced yield and quality.

Recent advances in the next generation genotyping technologies have helped to cut the cost and time while boosting genotyping precision. At the same time, phenotyping continues to be a barrier in establishing genotype-phenotype relationships (Yang et al., 2020). The introduction and evolution of phenomics in plant science occurred around 2010 with sensors to capture time series information and plant characteristics from digital images, which can ease the phenotyping bottleneck (Yang et al., 2020). Phenomics is the multidisciplinary study of high-throughput accurate acquisition and analysis of multidimensional phenotypes by using digital sensors to capture the morphological and physiological responses of plants (Kumar et al., 2016; Tardieu et al., 2017; Yang et al., 2020). Throughout plant growth and developmental life cycle, phenomics aids in the study of plant morphometry, physiology, leaf color, biomass, seed characteristics, spike number, growth rate and water use efficiency (Chen et al., 2014; Al-Tamimi et al., 2016; Guo et al., 2018; Misra et al., 2020; Elangovan et al., 2023). Researchers across the globe are targeting many plant traits to break the current plateau of the yield (Rauf et al., 2015; Neeraj et al., 2022). The harvest index is one of the most important traits as it links biomass accumulation and grain yield in cereals (Reynolds et al., 2017) and hence biomass is one of the key traits for crop improvement. Plant leaf area is an important conventional physiological trait used for plant developmental studies and leaf area index estimation (Wu et al., 2022). It is an essential parameter for assessing crop growth and is highly related to the crop biomass and yield (Qiao et al., 2019; Wu et al., 2022).

Manual estimation of plant biomass and shoot area gives accurate information but it is destructive, time and labour intensive, less throughput, and the accuracy is affected due to human errors (Buxbaum et al., 2022) thereby restricting its use in breeding and commercial contexts. Hand-gathered allometric approaches that connect plant volume and height data to biomass are time-consuming, arduous, and may not generalize (Pottier and Jabot, 2017). Also, by the conventional phenotyping, we could only get biomass and leaf area as a single point data, while the high throughput phenotyping empowered generation of time series biomass data (Rahaman et al., 2017; Song et al., 2021). Predicting biomass at multiple stages gives more insight into complex yield architecture (Buxbaum et al., 2022), crop phenotypic (P) and genotypic (G) along with G×E behaviour of plants (Xu, 2016; Van Eeuwijk et al., 2019). As biomass is a time-dependent variable in the plant life cycle, its non-invasive measurement at multiple time points is essential to dissect the complex plant growth characteristics and for its functional mapping (Wu and Lin, 2006; Li and Wu, 2010).

Non-invasive biomass estimation was mainly carried out, in the past, with a single sensor and very few image-based features (Campbell et al., 2015; Al-Tamimi et al., 2016; Rahaman et al., 2017; Asif et al., 2018). Researchers also have tried to predict biomass as linear function of projected area (Golzarian et al., 2011), multiple linear regression of different parameters, considering both the volume of the plants and their density (Busemeyer et al., 2013; Yang et al., 2014) and with four machine learning based biomass estimation from multiple sensor traits (Chen et al., 2018b). Many well established machine learning methods have been used earlier for various purposes, such as the prediction of gene expression patterns due to chromatin features (McLeay et al., 2012; Yang et al., 2014; Song et al., 2016), biomass (Chen et al., 2018b), and classification of the disease status of plants (Baranowski et al., 2015). Several models have been developed for in-house experiments to predict biomass of Arabidopsis (Arvidsson et al., 2011), barley (Bendig et al., 2015; Chen et al., 2018b), wheat (Golzarian et al., 2011; Parent et al., 2015), and rice (Yang et al., 2014; Campbell et al., 2015), but their reproducibility in other experiments has not been characterized. Also, researchers have tried to predict biomass at early stages (Golzarian et al., 2011; Chen et al., 2018b) which might not cover all the phenotypic variability of plant biomass. Machine learning model-based prediction of biomass and leaf area in wheat under controlled environment condition have not been reported yet.

So, we planned our experiment to develop a generalized robust protocol for non-destructive estimation of biomass and shoot area in wheat at peak vegetative stage by using open-source machine learning tools from the large number of image-based features and from multiple sensors which can be used to precisely predict plant biomass in future experiments by plant scientists. The objectives of this study were (i) to generate multi-experiment phenomics data from multiple sensors to predict plant biomass and shoot area at vegetative stage in wheat (ii) to select the best generic model for accurate prediction of fresh weight (FW), dry weight (DW) and shoot area (SA) by using open source machine learning tools (iii) to identify best surrogate i-Trait for FW, DW and SA. As a result, we screened a generalised model from a large set of machine learning models which considers traits derived from multiple sensors incorporating geometric features, RGB indices, colour class and NIR features covering major determinants of plant growth and also showing higher accuracy across experiments.

## Materials and methods

### Experimental design for biomass estimation

Two independent experiments were conducted in the Nanaji Deshmukh Plant Phenomics Centre (28°38’31.2”N, 77°09’39.6”E), New Delhi, India, during the winter seasons (Nov-April, Rabi) of 2018 and 2019. In both experiments, wheat germplasm lines and recombinant inbred lines (RILs) of Raj3765 x HD2329 were used. Seeds were sown in pots (0.19 m diameter, 0.4 m high, 15 L volume) containing uniformly filled soil (12.5 Kg per pot). Both experiments differed in genotypes and RILs to validate the model’s effectiveness in a wide range of biomass across experiments. Recommended dose of fertilizer (120-80-60 kg/ha N-P-K respectively) was applied to each pot. Well watered condition was maintained in all the pots and recommended weed, pest and disease control practices were followed. Plants were grown in the natural environment for proper growth and biomass accumulation, and shifted to the greenhouse in phase wise manner to capture the biomass at different vegetative stages (before booting). The idea to capture biomass before booting was due to the fact that after booting the ear creates erroneous result due to its variable weight than leaf and stem. The age of plants were ranging from 30 to 70 days after sowng at the time of imaging. This variability in plant age helped in capturing wider range of FW, DW and SA. Before imaging of the plant, ultra-low weight & solid polypropylene beads were applied just over the soil surface to about 5cm height to arrest direct evaporation from the soil surface, and also for easy segmentation of image. Three hundred plants in 2018 experiment and 154 plants in 2019 experiment were selected for image acquisition using the LemnaTec-Scanalyzer 3D automated phenotyping and imaging platform. Destructive sampling of plants was done to measure above-ground biomass FW (g), DW (g), and SA (cm^2^). The SA of the whole plant, along with stem and leaf, was measured by using LI-3100C (LI-COR, Lincoln, NE, USA) automatic leaf area meter.

### Image acquisition and processing

RGB and NIR images of the plants were taken using a commercial grade RGB (Prosilica GT6600, sensor: ON Semi KAI-29050, LemnaTec, GmbH, Aachen, Germany) and NIR camera (Gold eye P-032 SWIR Cool cameras, sensor: InGaAs, LemnaTec, GmbH, Aachen, Germany) using LemnaTec-Scanalyzer 3D software. Three different side views of RGB images (angles: 0°, 120°, 240°), and one top view RGB image of the plants were captured for each plant using the automated turning and lifting system inside the imaging unit. Three side views were considered, as it is hypothesized that the image from one direction cannot cover all the plant parts; besides, it helps increase the data points corresponding to one plant. NIR sensor captured one side view and one top view image for analysis. A uniform white background was maintained to increase the accuracy of separation between the background and foreground in the images.

Images were processed by the wheat image analysis pipelines developed in the commercial LemnaGrid software. Images were pre-processed to segment the image into foreground and background sections accordingly, and then feature extraction was done to produce a trait list. Extracted traits from the whole dataset were exported in CSV format *via* LemnaGrid and LemnaMiner functionalities, which were used for post-processing and statistical analysis. A detailed data set report is available in (**Supplementary Table 1**).

### Feature analysis and data transformation

After feature extraction, all the features were categorized into four groups: Geometrical features, colour class features, RGB-based indices and near-infrared features. Finally, these features are classified into two major categories namely architectural features (geometric features) and physiological features (colour class, RGB based indices and NIR features). Details of these features are available in **Supplementary Table 1**. These features were specified by considering the type of imaging sensors (RGB and NIR) and object orientations (side and top views). All the traits were curated for redundancy, processing error, outliers, and non-informativeness by both statistical approach (Multicollinearity removal, poor heritability, etc.) and manual curation. We kept as much variation and informative features as possible to improve the model accuracy for biomass estimation.

Each experiment dataset was transformed into matrix Xn×m where “n” is the number of plants and “m” is the number of phenotypic traits. Plants represented rows, and different traits represented columns. All missing value plants were discarded for reduction of data analysis complicacy. Before applying regression models, all datasets were normalized as described by (Chen et al., 2018a).

### Phenotypic data interpretation and visualization

A phenotypic similarity tree was used to see the correlation between all the traits and the similarity between experiments. Principle component analysis was performed on the transformed data matrix Xn×m in the same way as described by (Chen et al., 2014) for all the experiments. Both correlation and PCA analysis was done in R software (R Core Team, 2021). All visualization graphs were produced using “ggplot2” package in R software (R Core Team, 2021).

### Modelling for predicting plant biomass

After i-Trait selection and phenotypic analysis, the next part was to fit the selected data into the model to predict FW, DW, and SA. We used the open-source tool “HTPmod” for modelling (Chen et al., 2018a). In HTPmod (Shiny framework-based application), the module *predMod* contains 16 models constructed with 16 different machine learning methods to regress input features to output traits of interest. The description and details of all the models is available in **supplementary data sheet 1**. We used the default hyperparameters applicable for different machine learning models present in the HTPmod application. All the model parameters were controlled using respective R package (Given in **supplementary data sheet 1**). Also, for additional tuning functionality of “caret” R package was used.

### Evaluation of the models performance

Model performance was evaluated using k fold cross-validation method and N-times randomization, where we assigned k and N to 10. So, we adopted a 10-fold cross-validation strategy and ten times randomization for model evaluation by considering the average value. The data set was randomly divided into a training set of 90% of plants and a testing set of the remaining 10% of plants. Then each model was run to predict FW, DW, and SA for the testing data, and then the predicted biomass was compared with the manually measured FW, DW, and SA.

All the regression models were evaluated by the Pearson correlation coefficient (PCC; *r*), the coefficient of determination (R^2^) and the root mean squared relative error of cross-validation (RMSRE) between the predicted and observed values (Chen et al., 2018a).

## Results

### i-Trait extraction and characterization

**Figure 1:**
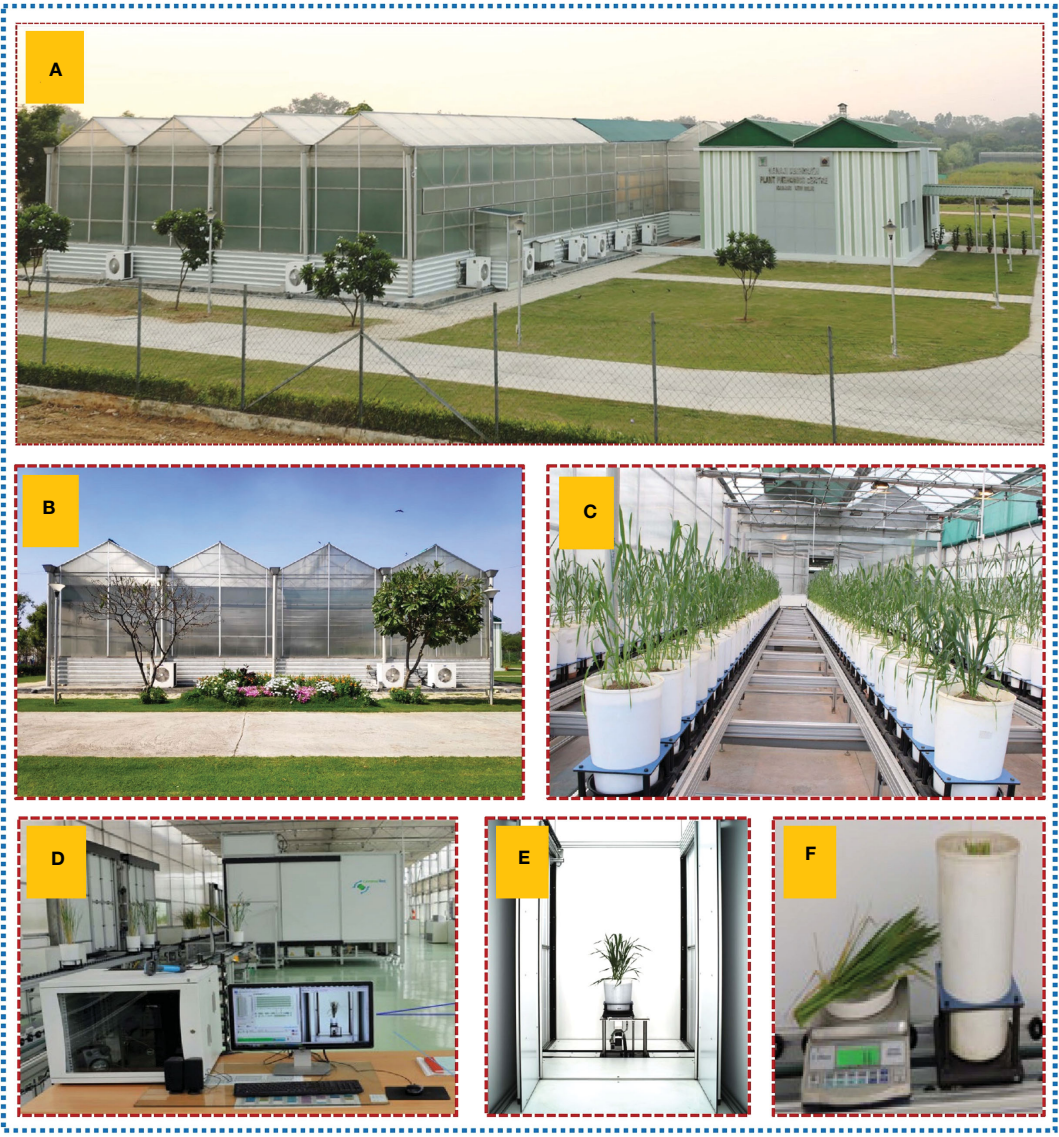
Pictorial representation of experimental site and setup. **(A)** Nanaji Deshmukh Plant Phenomics Center (NDPPC), Indian Council of Agricultural Research–Indian Agricultural Research Institute, New Delhi, India. **(B)** Four climate controlled green houses present within the facility. **(C)** Ongoing wheat plant experiment for wheat non invasive biomass prediction. **(D)** LemnaTec system controller computer for image acquisition and processing. **(E)** RGB, side view image of wheat plant inside imaging chamber. **(F)** Destructive sampling for biomass measurement.

**Figure 2:**
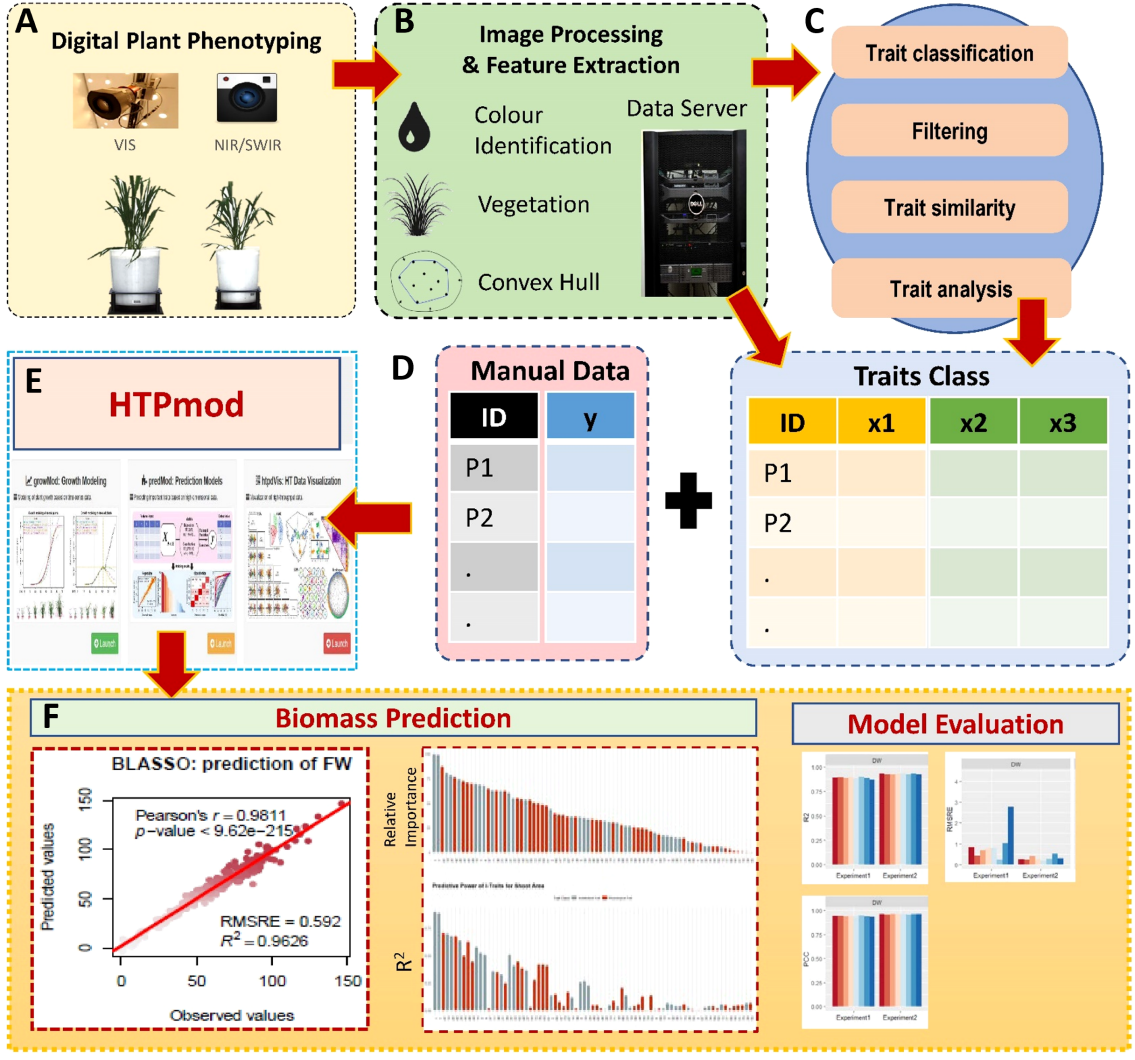
Data analysis and modelling pipeline for biomass prediction by i-Traits. **(A)** Digital imaging based plant phenotyping by visible (or color) and near-infrared sensors (NIR). **(B)** Image datasets were saved in data server and processed through LemnaGrid for feature extraction. **(C)** Phenotypic data were subjected to quality check to remove low-quality data and classified into two categories such as architectural and physiological traits (Commonly referred as i-Traits). **(D)** All the i-Traits were described by per plant basis and combined with manually measured data such as fresh weight (FW), dry weight (DW) and shoot area. **(E)** Now the data matrix with all i_traits and manual traits were used to predict biomass by HTPmod (a shiny based application) using eight machine learning method developed by Chen et al. (2018a). **(F)** The results of aal the models were interpreted by R^2^, PCC and RMSRE value.

We analysed two image datasets, 1800 images (Experiment 1, 2018) and 924 images (Experiment 2), collected from 300 (Experiment 1) and 154 plants (Experiment 2, 2019), respectively. An overview of the experimental site and experiment is shown in **Figure 1**. Each plant was imaged at a single time point by RGB (One top view and three side view images at 0°, 120° and 240° angle) and NIR sensor (One top view and one side view), then plants were harvested to measure FW) and SA immediately, and oven dried to record DW. To increase the variability in biomass range, we conducted our experiment with germplasm and RILs with wider variability in biomass, and phenotyping at different plant growth stages. All the images retrieved from the server and processed by the LemnaGrid image analysis pipeline, which was modified explicitly for mid to large-sized important cereals such as wheat (challenging due to its narrow leaf and compact character), resulting in nearly 200 phenotypic traits extracted from images of each plant (**Figures 2A, B**). After quality control such as outliers, multicollinearity and manual checking of all the extracted traits, we selected 77 i-Traits (**Figure 2C**) which were divided into two major categories of traits such as architectural (35 traits) and physiological (42) traits (**Supplementary Table 1**).

### Phenotypic profile analysis of plants in both experiments

**Figure 3:**
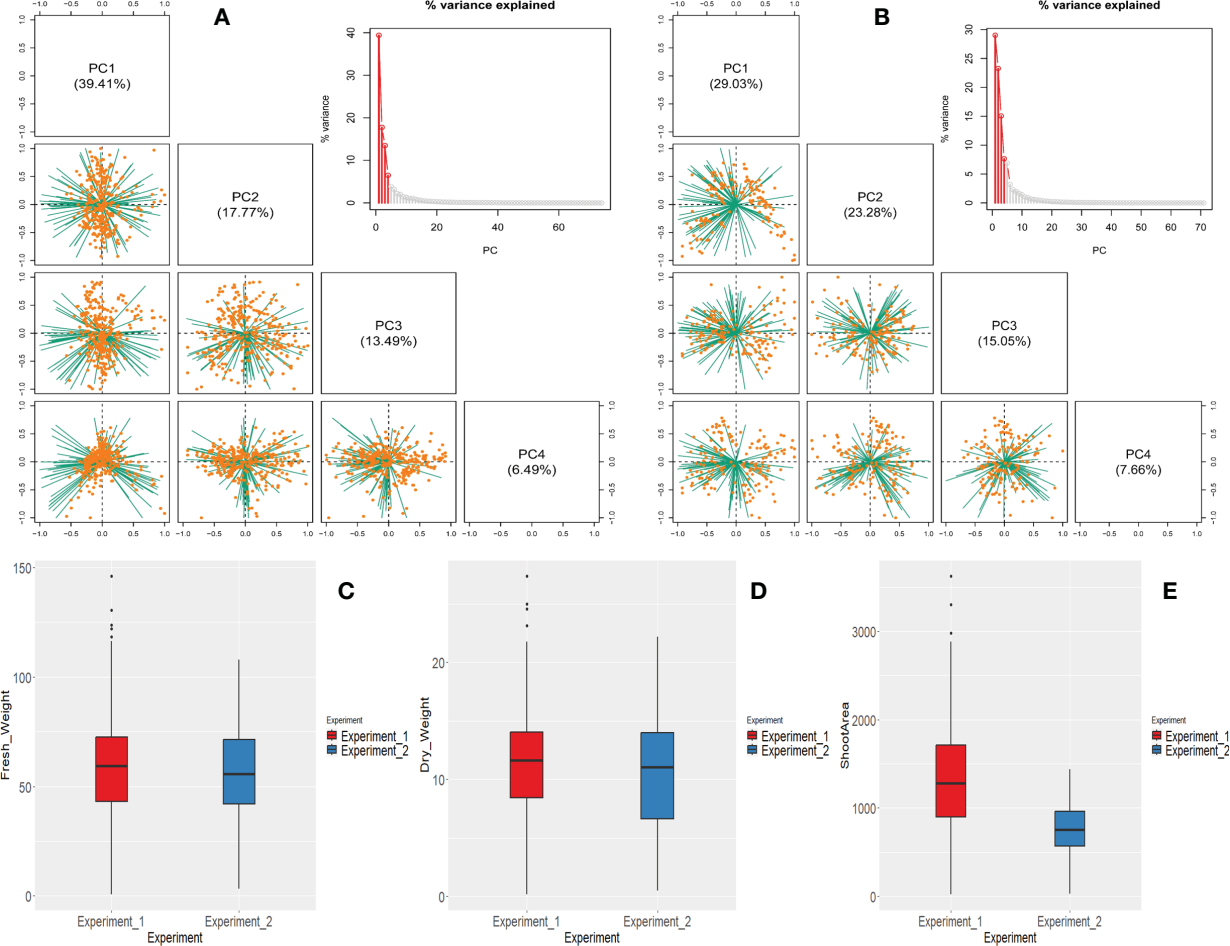
Characterization of all the i-Traits by phenotypic analysis for both experiments (Experiment 1 and Experiment 2). **(A, B)** Principal component analysis of all the i-Traits for experiment 1 and experiment 2 respectively. Four PCs represented here which captured approximately all the variation in the data. Variance proportion explained by the PCs is shown in parentheses. Individuals are represented by orange dots and traits are represented by green lines. **(C–E)** Differences in the FW, DW and shoot area across experiments represented by box plot analysis.

We observed broader range of phenotypic values in all the traits in both experiments. The phenotypic value of FW, DW, and SA of experiment 1 ranged 0.6-145.86 g, 0.15-27.35g and 18.43-3622.25 cm^2^, respectively. In the experiment 2, the FW, DW, and SA ranged 3.3-107.8 g, 0.5-22.2 g, and 26.15- 1434.01 cm^2^, respectively (**Figures 3C-E**).

In both experiments, 77 selected i-Traits were analysed. Principal component analysis (PCA) was carried out for both experiments to see the global phenotypic variation present in the population. The top 4 principal components (PCs) of experiment 1 and 2 accounted for 76.56% and 75.02% of the total phenotypic variation explained by i-Traits. The first two PCs clearly distinguished the experiments as the first two PCs of experiment 1 and 2 accounted for 39.41%, 17.77% and, 29.03%, 23.28%, respectively (**Figures 3A, B**).

**Figure 4:**
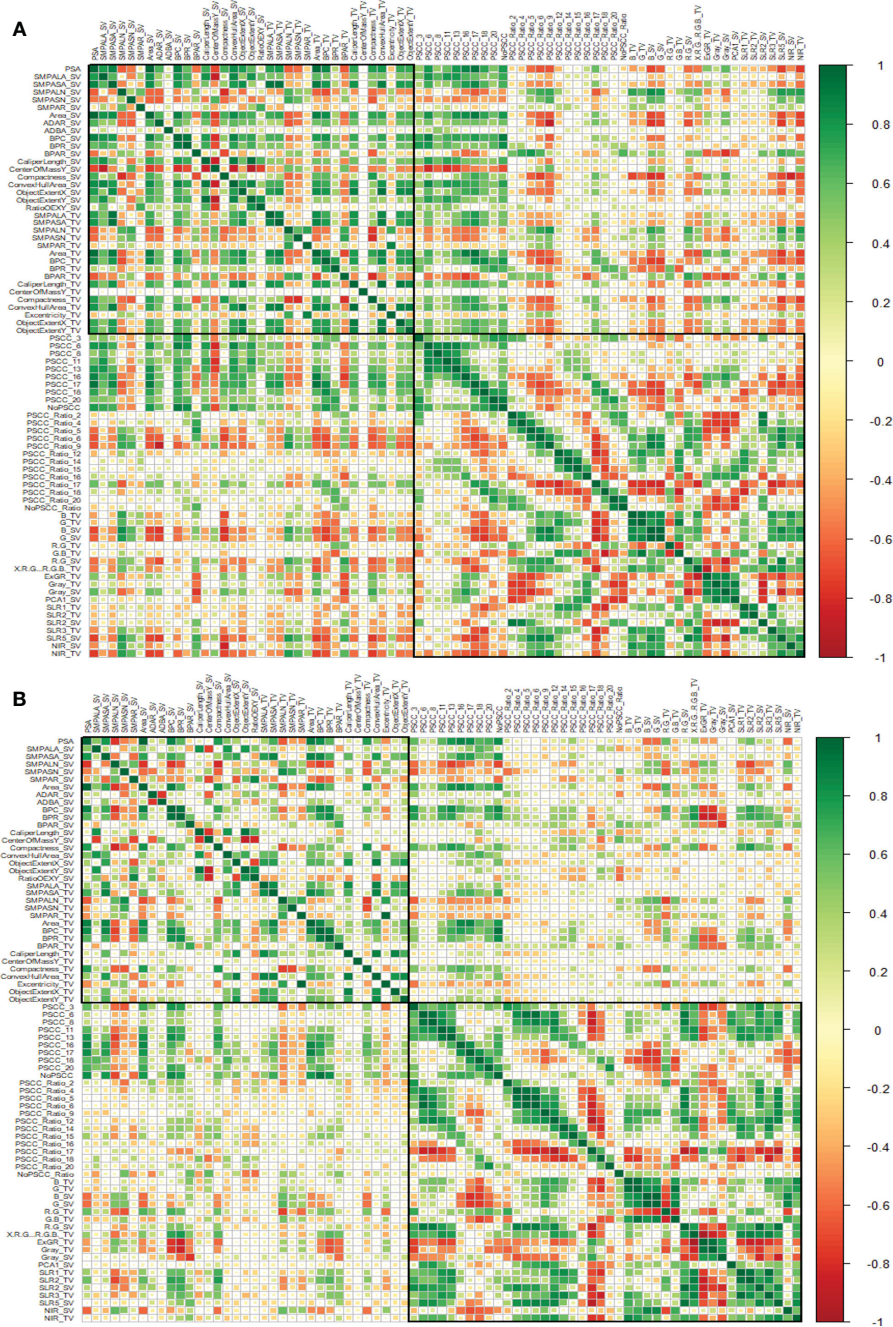
Assessment of trait similarity between i-Traits across experiments. **(A)** Canonical correlation analysis of i-Traits based on experiment 1 (Top) and **(B)** experiment 2 (Bottom). Heatmap plot is organized by both architectural and physiological traits represented by two highlighted boxes. Top box represents Architectural traits and bottom box represents physiological traits.

To access the patterns of trait correlations, we performed the trait similarity analysis based on canonical Pearson’s correlation coefficient (**Figures 4A, B**). We observed that the patterns of correlation were similar in both experiments however i-Traits were more correlated in experiment 1 than in experiment 2 as suggested by the intensity of colour in **Figures 4A, B** but traits across experiments shows similar correlation with each other. Also, the correlation of architectural traits with physiological traits was less, and traits were both positively and negatively correlated in the two experiments. Correlation among the physiological traits as higher than the correlation among the architectural traits.

### Phenotypic association of i-Traits with FW, DW and shoot area

**Figure 5:**
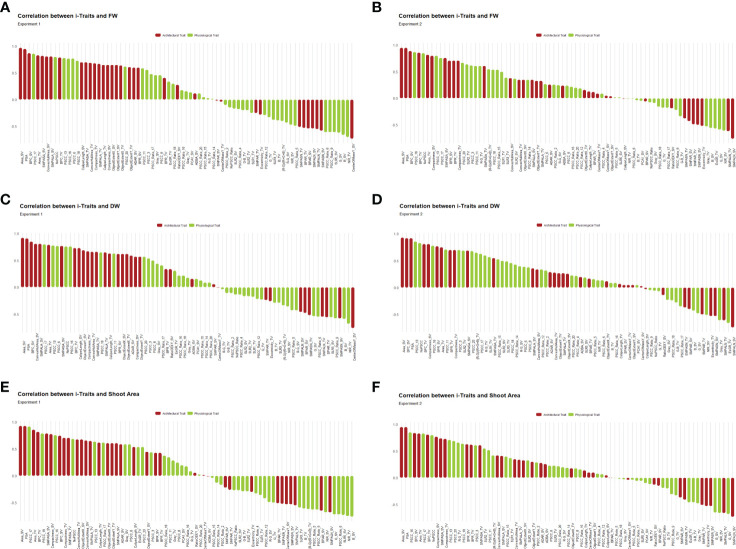
Pearson’s correlation coefficients (PCC) in each experiment were calculated to assess the relationship between i-Traits and manual traits. The PCC were consistent in both experiments and most of the features were having more than 0.5 positive or negative correlation coefficients with FW, DW and shoot area. **(A, B)** PCC between FW and i-Traits in experiment 1 and 2 respectively. **(C, D)** PCC between DW and i-Traits in experiment 1 and 2 respectively. **(E, F)** PCC between shoot area and i-Traits in experiment 1 and 2 respectively.

We further evaluated the association of i-Traits with the manual traits in both experiments to see the relation between i-Traits and manual traits and to observe the biological relevance of predicting biomass from digital image-derived parameters (**Figure 5**). The direct relatedness of architectural traits with biomass is well reported in literature and the physiological traits that we included here having some unique feature that have not been reported earlier such as RGB colour class and RGB indices. The use of physiological traits in biomass estimation is to incorporate the additional properties presented by NIR grey value (water status of plant), RGB colour class (greenness of different plant pixels) and RGB indices (reported vegetation indices). The PCC of the i-Traits with FW, DW and SA in experiment 1 ranged from -0.73 to 0.97, -0.76 to 0.92, and -0.76 to 0.93, respectively. While in the case of experiment 2, the FW, DW and SA correlations with i-Traits ranged from -0.76 to 0.95, -0.74 to 0.93 and -0.73 to 0.95, respectively. Architectural traits had a higher correlation with manual traits, than physiological traits. Among all the traits, PSA and Area_SV had a correlation of >0.9 for FW, DW and SA in both experiments. As expected, both boundary point count and compactness of side view images are also highly correlated with the manual traits. This explains that the biologically relevant traits, such as area and architecture, are highly related to biomass traits. We also found that physiological traits such as colour class correlated with biomass traits. NIR_SV was negatively correlated with FW, DW and SA ranging from -0.36 to -0.68 in both experiments. This also suggests that physiological traits not directly measured as plant architectural traits can also be used as biomass predictors. Indices derived from the mean blue, green and red values of RGB images also correlated with biomass, but the correlations were relatively lower than other traits.

### Modelling of plant biomass using machine learning methods

HTP is particularly meaningful in dissecting complex genetics of biomass development in plants. The relationship between i-traits and above-ground shoot biomass accumulation were analysed by digital phenotyping data containing structural (e.g., architectural traits) and physiological traits (e.g., colour class, RGB based indices and plant moisture content as reflected by NIR-intensity traits). The results about i-Traits and manual traits suggested that these i-Traits can be very useful in predicting plant biomass-related traits.

To develop the model by machine learning (ML) methods, we used the available open-source tools. We used the *predMod* module from the HTPmod, a R program based shiny application. We used 16 ML methods available in *predMod*. From the 16 models, we found eight models that were consistent for all the manual trait estimations in both the experiments. The selected models were Bayesian LASSO, Bayesian regularized neural networks (BRNN), Lasso and elastic-net regularized generalized linear models (GLMNET), Gaussian process with the polynomial kernel (GP-Poly), multivariate adaptive regression splines (MARS), random forest (RF), ridge regression (RIDGE) and Support vector machines with linear kernel (SVM-Linear).

### Biomass estimation model performance evaluation

**Figure 6:**
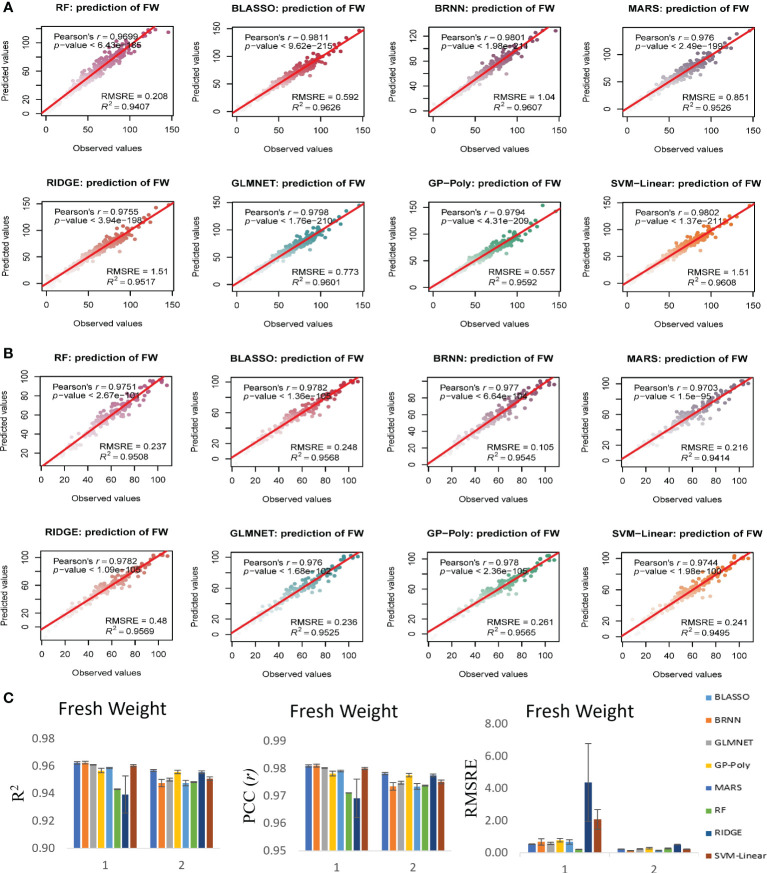
Non destructive estimation of above ground plant biomass (AGPB) with i-Traits, using *predmod* in HTPmod. Scatter plots of observed FW vs predicted FW values using 8 prediction models based on machine learning methods BLASSO, BRNN, GLMNET, GP-Poly, MARS, RF, RIDGE and SVR. The prediction models were evaluated by Pearson’s correlation coefficient (*r*), its corresponding *P*-value, R^2^ and RMSRE. **(A, B)** Prediction of FW in experiment 1 and 2 respectively. **(C)** Evaluation of the model performance of each regression model used for AGPB prediction.

**Figure 7:**
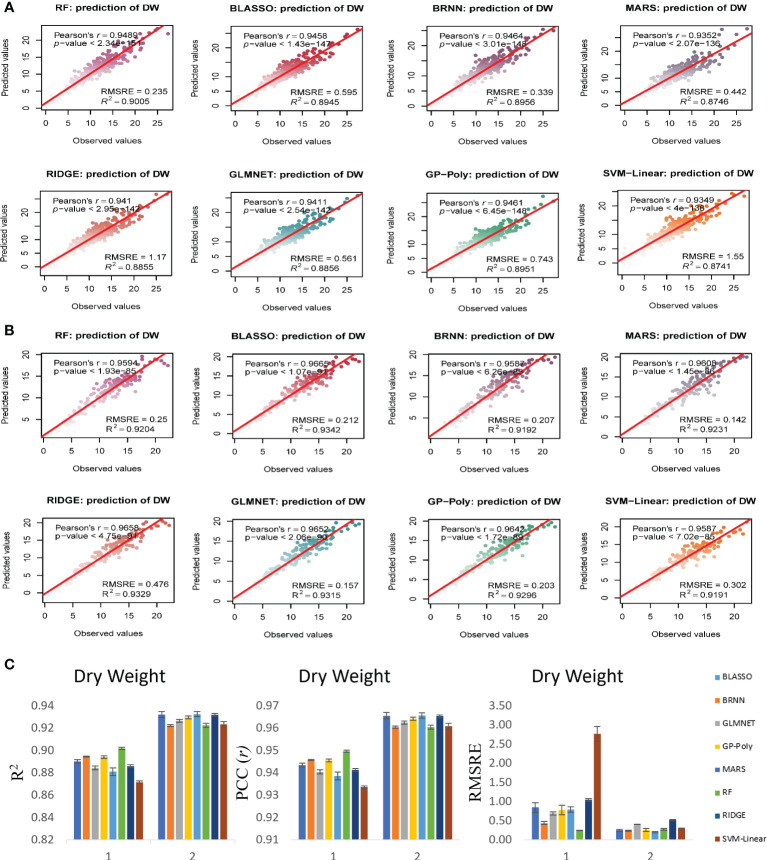
Non destructive estimation of DW by using i-Traits. **(A, B)** Prediction of DW in experiment 1 and 2 respectively. **(C)** Summary of the predictive power of each regression model.

**Figure 8:**
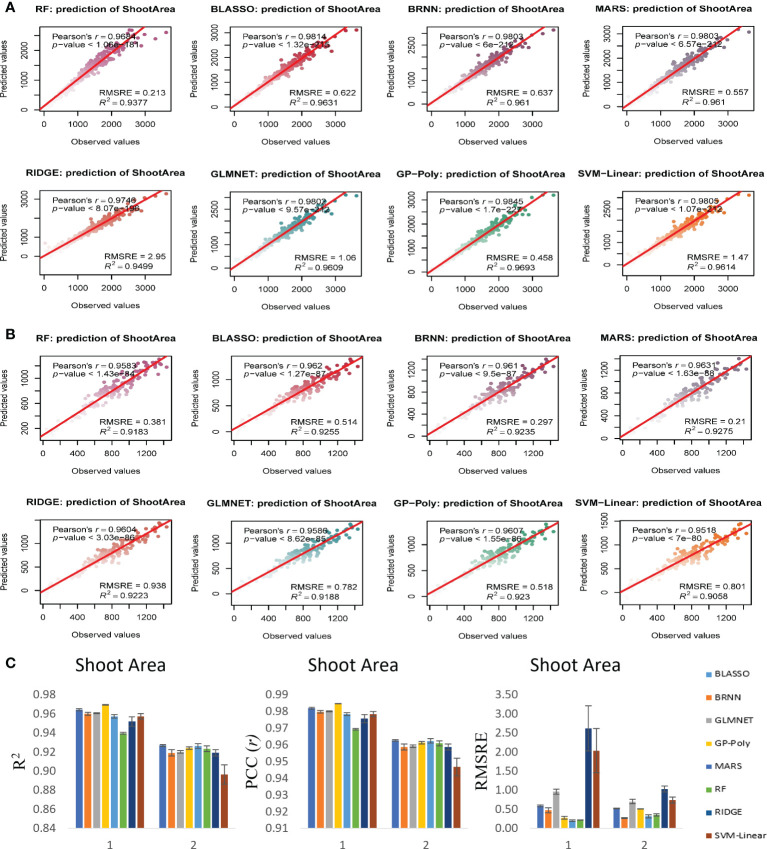
Non destructive estimation of shoot area by using i-Traits. **(A, B)** Prediction of shoot area in experiment 1 and 2 respectively. **(C)** Summary of the predictive power of each regression model.

Since our aim was to produce a more generalized model that can use genotypes and RILs to predict the biomass from image traits over multiple time points, the models were tested in both experiments to validate their performance and to evaluate their generalized nature. Analysis was performed with all 16 models, from which eight models that performed better for all the traits irrespective of experiments were selected. Results of all the 16 models showed that the R^2^ for FW prediction was between 0.84 to 0.96, DW prediction was between 0.79 to 90 and SA prediction was 0.85 to 0.97 in experiment 1 (**Supplementary Data sheet 1**). In experiment 2 the R^2^ for FW prediction was between 0.88 to 0.96, DW prediction was between 0.86 to 93 and SA prediction was 0.86 to 0.93 (**Supplementary Data sheet 1**). We found that eight models performed relatively better than other eight models. Bayesian Generalized Linear Model (BGLM), Gradient Boosting Machine (GBM), Generalized Linear Model (GLM), Gaussian Process with Radial Kernel (GP-Radial), K-Nearest Neighbors (KNN), Least Absolute Shrinkage and Selection Operator Regression (LASSO), Multivariate Linear Regression (MLR) and Support Vector Machines with Radial Kernel (SVM-Radial) performed with relatively less accuracy than other eight models namely Bayesian LASSO, Bayesian regularized neural networks (BRNN), Lasso and elastic-net regularized generalized linear models (GLMNET), Gaussian process with the polynomial kernel (GP-Poly), multivariate adaptive regression splines (MARS), random forest (RF), ridge regression (RIDGE) and Support vector machines with linear kernel (SVM-Linear). BGLM, GBM, GLM, GP-Radial, KNN, LASSO, MLR and SVM-Radial had R^2^ value for prediction of FW ranged from 0.84 to 0.90, for DW ranged from 0.79 to 0.87 and for SA ranged from 0.85 to 0.95 in experiment 1 (**Supplementary data sheet 1**). At the same time BLASSO, BRNN, GLMNET, GP-Poly, MARS, RF, RIDGE and SVM-Linear had higher R^2^ value of 0.94 to 0.96 for FW, 0.87 to 0.90 for DW and 0.93 to 0.96 for SA prediction in experiment 1 (**Figures 6**–**8**). Similar condition was there in experiment 2 also. In experiment 2, BGLM, GBM, GLM, GP-Radial, KNN, LASSO, MLR and SVM-Radial had R^2^ value of 0.88 to 0.92 for FW, 0.86 to 0.89 for DW and 0.86 to 0.90 for SA prediction (**Supplementary data sheet 1**). While BLASSO, BRNN, GLMNET, GP-Poly, MARS, RF, RIDGE and SVM-Linear had higher R^2^ value of 0.94 to 0.96 for FW, 0.91 to 0.93 for DW and 0.90 to 0.93 for SA prediction in experiment 2 (**Figures 6**–**8**). We also noticed that these eight models performed better than normal multivariate regression (MLR) model. All eight selected models BLASSO, BRNN, GLMNET, GP-Poly, MARS, RF, RIDGE and SVM-Linear performed relatively similar in terms of estimation accuracy, while we confined the results to select four best-suited models for our experiments namely BLASSO, BRNN, GLMNET and GP-Poly that were with less RMSRE value and with similar estimation accuracy across experiments.

In case of FW estimation, R^2^ and *r* value ranged from 0.95 to 0.96 and 0.97 to 0.98 in the two experiments for BLASSO, BRNN, GLMNET and GP-Poly models (**Figure 6**). So, this represents the generalized nature of the estimation, as almost all four models showed similar results. DW’s estimation also showed promising results as the R^2^ and *r* value ranged from 0.89 to 0.93 and 0.94 to 0.96 in the two experiments (**Figure 7**). In SA estimation, R^2^ and *r* value ranged from 0.91 to 0.97 and 0.95 to 0.98 in both the experiments (**Figure 8**). As per these four models, Experiment 1 had slightly higher estimation accuracy than experiment 2 for FW and SA estimation but in the case of the DW, experiment 2 had better performance than experiment 1. RMSRE values were relatively lower in experiment 2 than in experiment 1 in most of the parameters but the difference was not large.

By observing the estimation ability of the models by R^2^ and *r* and RMSRE we found out BLASSO, BRNN, GLMNET and GP-Poly were relatively better-performing models in all the traits and across all the experiments. Selecting a single model was interesting due to the relatively similar and better estimation accuracy across models. Nevertheless, BLASSO performed better in all the trait estimations in all the experiments with the highest R^2^ and lowest RMSRE values. The R^2^ of Bayesian Least Absolute Shrinkage and Selection Operator BLASSO for FW prediction was 0.96 (both year experiments), for DW prediction was 0.90 (Experiment 1) and 0.93 (Experiment 2) and for SA prediction 0.96 (Experiment 1) and 0.93 (Experiment 2). Also, the RMSRE of BLASSO for FW prediction was 0.53 (Experiment 1) and 0.24 (Experiment 2), for DW prediction was 0.85 (Experiment 1) and 0.25 (Experiment 2) and for SA prediction 0.59 (Experiment 1) and 0.53 (Experiment 2).

### Evaluation of i-Traits for predictive power and relative importance

**Figure 9:**
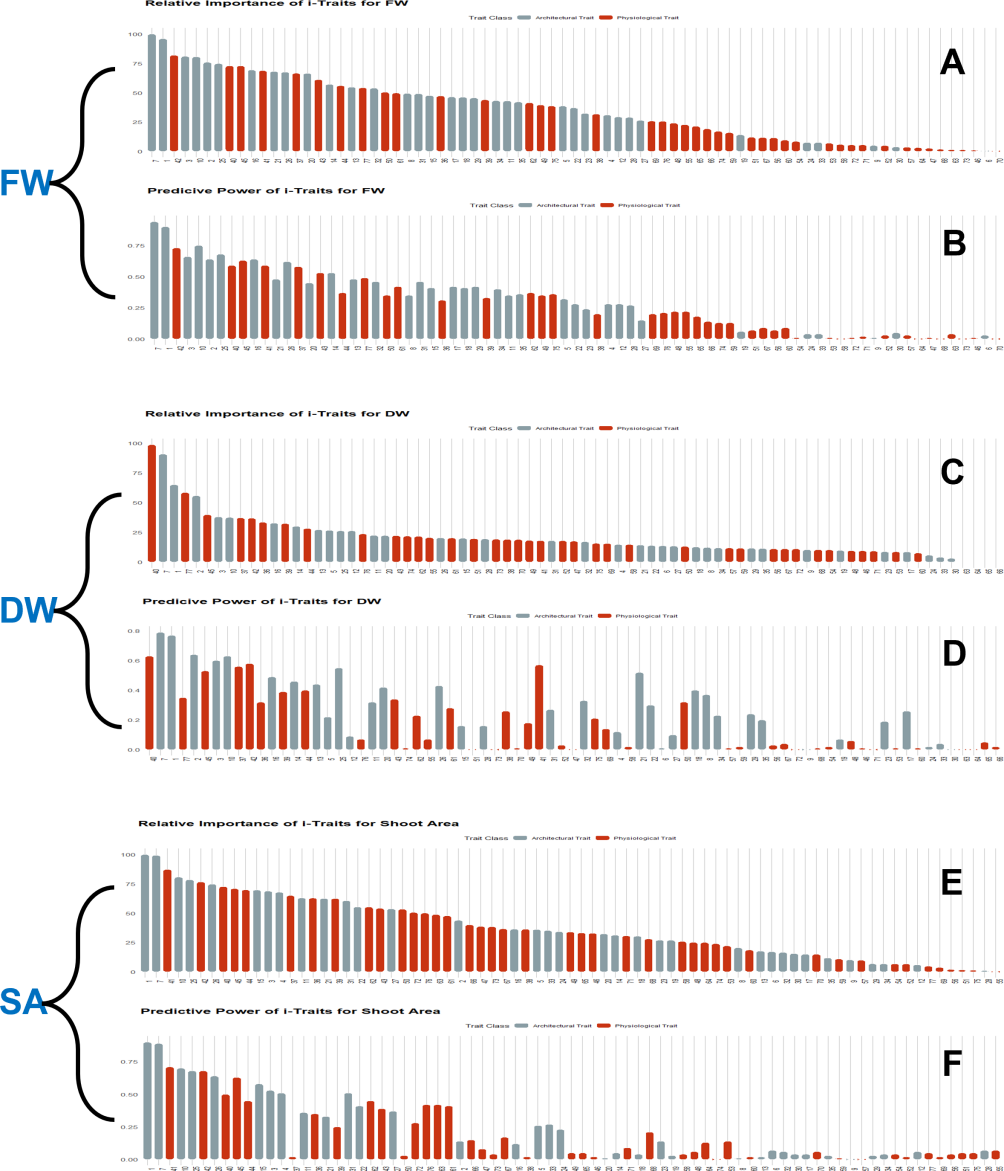
Estimation of predictive power and relative importance of features (i-Traits) used in regression models. Bayesian-LASSO (BLASSO) model was used for wheat FW, DW and shoot area prediction, using i-Traits from two independent experiments. But as both experiments performed relatively similar with respect to prediction power and relative importance, experiment 1 information is presented here for the ease of understanding. **(A, C, E)**. Relative importance of i-Traits used for FW, DW and shoot area prediction respectively. **(B, D, F)** Predictive power of i-Traits used for FW, DW and shoot area prediction respectively.

For each i-Trait, the predictive power and relative importance were calculated by the degenerate model using the *predMod* module. In both experiments, the predictive power and feature importance of i-Traits were similar, so here we explained the individual capability of each i-Trait as a predictor of biomass in experiment 1 (**Figure 9**) and experiment 2 (**Supplementary data sheet 1**). Both predictive power and relative importance of most architectural traits were higher than physiological traits. The top 10 important features contained architectural and physiological traits, but more architectural traits were present than physiological ones. PSA, Area_SV, and BPC_SV have higher predictive power and relative importance among all the i-Traits for FW, DW and SA. Area_SV had R^2^ of 0.94 for the estimation of FW, which was highest than the DW (R^2^ of 0.79) and shot area (R^2^ of 0.89) estimation. Among physiological traits, PSCC_17, 16, 13, NIR_TV and SV, B_SV, G_SV, SLR5_SV, Gray_TV, etc., had higher predictive power and relative importance than other physiological traits. NIR_TV and NIR_SV also had significant predictive power and relative importance ranging from ~0.1 to 0.5 (R^2^) and ~5 to 59% (Inclusive MSE) relative importance. The best predictor i-Trait for FW and DW prediction was Area_SV and for SA prediction was projected shoot area. These results give deep understanding into major determinants of plant biomass and also suggests that along with architectural traits, physiological traits also help to improve estimation accuracy and are determinants of plant growth.

## Discussion

Aided by multiple imaging sensors and computer vision optimization by machine learning methods, high throughput phenotyping has emerged as a major technology helping to dissect plant biological characters unseen before (Kumar et al., 2016; Singh et al., 2016; Strock et al., 2022). So, gathering multi-dimensional data over multiple time points at multiple organ levels is the critical component of HTP, which has boosted crop improvement programs (Roth et al., 2021; Pérez-Valencia et al., 2022). Harvest index has been a critical trait for cereal breeders for decades in crop improvement programs, which is closely related to the ability of the plant to accumulate plant biomass and convert it into yield (Chao et al., 2019; Porker et al., 2020). Since, already we have reached a plateau in improving harvest index, for further improvement in yield, we must improve biomass. We need to characterize accurate biomass of plants at different stages to identify genes working at different phenological stages for biomass production (Chang et al., 2019; Chen et al., 2020; Rabab et al., 2021). Also, data at multiple time points are needed for the functional mapping of plant biomass accumulation. Functional mapping can be a powerful tool to reveal transient and deterministic quantitate trait loci (QTLs) for a complex trait such as biomass (Wu & Lin, 2006; Camargo et al., 2018; Jiang et al., 2019; Lyra et al., 2020). Plant leaf area is a critical physiological parameter which determine the ability of plant to produce economic yield and also it helps to determine the plant leaf area index which is an important conventional physiological trait used for plant developmental studies (Wu et al., 2022). Leaf area index (LAI), is a critical parameter of wheat growth, can provide dynamic information during wheat growth phases and closely associated with crop biomass and yield. So, shoot area estimation by non-destructive methods will definitely help to the plant science community in the future. As per our knowledge no work has been done particularly for image-based shoot area estimation by validating with actual shoot area in wheat as it has a compact plant architecture. Traditionally, biomass measurement has been done by destructive methods, which is laborious and time-consuming. Also, one of the other major lacuna was the inability to take data points at multiple time scales (Tackenberg, 2007; Buxbaum et al., 2022). Therefore, to address this major bottleneck, automated, non-destructive biomass estimation by digital imaging method is gaining importance since the evolvement of high throughput phenotyping (Golzarian et al., 2011; Rahaman et al., 2017; Chen et al., 2018b; Buxbaum et al., 2022).

Several studies in the recent past have developed different models for biomass estimation, but their applicability in other in-house experiments has not been tested widely. Golzarian et al. (2011) and Chen et al. (2018b) predicted biomass in wheat at the age of 15 to 43 days after sowing and in barley at the age of 27 to 58 days after sowing respectively, where crop biomass is not very high and overlapping of leaves are less. But it is challenging to predict the biomass of wheat plant at peak vegetative growth. Also, different crops pose different plant architecture such as leaf distribution, tiller numbers overlapping of leaves etc which affects the biomass prediction model accuracy. So, a robust and open-source model that excludes the need for repeated destructive measurement is the need of the hour for any high throughput phenotyping facility worldwide. To address this, we conducted an experiment to estimate wheat biomass and related traits non-destructively in the largest phenomics facility in India, Nanaji Deshmukh Plant Phenomics Centre (NDPPC). We aimed to develop a pipeline to predict plant FW, DW and SA non-destructively by open-source tools, which can be used in future experiments by different researchers. We conducted two experiments in consecutive years to see if the models work equally well in different datasets.

The selection of predictor variables for the estimation of a trait is necessary. In the past, single i- trait based biomass estimation (Tackenberg, 2007; Golzarian et al., 2011; Campbell et al., 2015) and modified experiments with multiple traits have been carried out (Yang et al., 2014; Rahaman et al., 2017; Chen et al., 2018b). Previous studies have suggested that combining multiple traits such as vegetation indices and plant-height information can improve biomass estimates (Bendig et al., 2015; Han et al., 2019). Reports have shown that multiple traits and multi-sensor based estimation of biomass have more accuracy and biological meaning (Chen et al., 2018b) which includes different categorical features such as geometric or architectural traits, colour based and NIR based physiological traits. NIR reflectance have been reported to determine water status of plants (Neilson et al., 2015; Jin et al., 2017). As two plants having same plant architecture but different water status will differ in their fresh weight due to the differences in water status. By taking a leaf out of the literature review of non-destructive plant biomass, we selected both RGB and NIR-based traits for our study. We included architectural, colour class based, mean red, blue and green-based indices and mean grey values to improve the estimation accuracy and make a robust model. We selected 77 high-quality i-Traits by removing redundant, non-informative traits using statistical and manual methods. Both experiments 1 and 2 have differed in phenotypic responses as accessed by principal component and box plot analysis (**Figure 3**) which emphasizes the independent nature of experiments. So, this will help in understanding the generalised nature of the models across experiments.

We predicted FW, DW and SA using 77 i-Traits by 16 machine learning methods which was available in open-source tool “HTPmod”. To validate the results, we also ran all the models in another dataset (experiment 2) with the same i-Traits for the estimation of FW, DW and SA. The estimation accuracy was consistent in both experiments which suggests that all those models were generalized in nature and selecting a particular model will not discriminate much accuracy. By observing the predictive power, PCC and RMSRE, we found eight models to be performing relatively similar with higher estimation accuracies. We included multivariate adaptive regression splines (MARS), random forest (RF) and support vector machine-Radial regression (SVR) in our selected eight models as they had accurate biomass predicting ability (Chen et al., 2018b). All the predictive models worked accurately for FW, DW and SA in both experiments. But we found that eight models namely BGLM, GBM, GLM, GP-Radial, KNN, LASSO, MLR and SVM-Radial performed relatively poor than other eight models namely BLASSO, BRNN, GLMNET, GP-Poly, MARS, RF, RIDGE and SVM-Linear. Here it is interesting to see that simple multivariate linear regression was in the relatively poor performing group which suggests that use of complex machine learning model improved the accuracy of prediction than simple MLR model (Chen et al., 2018b). The main reasons for the difference in the prediction accuracy of different machine learning models depends upon factors such as model assumption parameters, model architecture, overfitting of model, sensitivities to extreme values, collinearity present within the data set, complexity of the model and number of samples (Ogutu et al., 2012; Arruda et al., 2015; Esposito et al., 2019; Adak et al., 2021). From all the 16 models four models performed relatively poor than other models namely BGLM, GP-Radial, KNN and SVM-Radial. BGLM model is less suited for small scale data and affected by multicollinearity (Koehrsen, 2018), GP-Radial has problem of scalability, representational power and targeted optimisation (Hensman et al., 2013; Krauth et al., 2016), KNN mostly used on classification and detection based problems and has limitations of poor performance under high dimensionality and sensitive to extreme values (Uddin et al., 2022) and SVM-Radial has sensitivities to extreme values, collinearity and more helpful for classification problems. Although these are very good machine learning algorithms cited in literature and worked well under different datasets but in our dataset and type of data, they performance were hampered.

However, for general use and model selection purposes, we made the more stringent model selection with respect to robustness, predictive power, PCC and RMSRE for ease of use in future experiments. The four models BLASSO, BRNN, GLMNET and GP-Poly were consistent across the experiments in FW, DW and SA estimation with relatively similar predictive power, PCC and RMSRE value. These four models had higher R^2^ and PCC value and lower RMSRE value than other models. The estimation accuracies of these models were at par with the accuracy that have been reported (Neumann et al., 2015; Rahaman et al., 2017; Chen et al., 2018b) in literature and also the reproducibility of the accuracy was validated by the results from our second experiment. Also, the validation of actual leaf area to predicted SA has been a unique feature of this undertaken research. Among all these models we found BLASSO to be the best model in terms of high accuracy and low RMSRE value for all the biomass and related trait prediction and was also stable and interpretable across experiments. BLASSO had highest R^2^ value and PCC value for both year experiment and lowest RMSRE value among all models. BLASSO is a popular high dimensional data analysis method. It can perform regularization and variable selection at the same time. This can increase the precision of predictions and interpretation of a problem (Vasquez et al., 2016). It has major advances in terms of assumptions regarding the sample distribution as it is independent of normality of sample distribution, very efficient in handling large data set and dimensionality also helps to overcome underfitting of model data. So, with the help of this model along with multi-sensor dataset, we were able to predict FW, DW and SA accurately in wheat.

Our results showed that by using open-source tools, we can predict FW, DW and SA of wheat plant accurately in any in-house experiment. The estimation accuracy of all the traits, such as FW, DW, and SA, across both experiments was consistent with past studies (Golzarian et al., 2011; Campbell et al., 2015; Neilson et al., 2015; Neumann et al., 2015; Rahaman et al., 2017; Chen et al., 2018b). It also reflected how individual trait impact estimation by analysis of the relative importance and predictive power of each trait. The critical features such as PSA, Area_SV, BPC_SV, PSCC_17, 16, 13, NIR_TV, SV, B_SV, G_SV, SLR5_SV and Gray_TV, which had consistently higher predictive power and more significant correlation with FW, DW and SA can be used as a surrogate for biomass accumulation and dissection of their genetic basis in crop improvement programs. As biomass is a complex trait showing both spatio-temporal variations upon different environments, its characterization can be done using the above-said i-Traits and models effectively at multiple time points.

There was no major limitation of this study but found some interesting challenges which can be addressed by conducting further research. This study was conducted in the controlled greenhouse conditions so the models selected here have to be tested to predict the biomass under field condition to check the generalised nature of the models. Increase in number of samples and testing of models in different abiotic stress conditions will give deeper insights into biomass modelling. As we have witnessed increase in prediction accuracy was achieved by multiple sensor data, use of advanced hyperspectral sensor data and LIDAR sensor data in further research can give new insight into prediction accuracy of machine learning models.

## Conclusion

We developed the biomass and leaf area estimation model using the Bayesian Least Absolute Shrinkage and Selection Operator (BLASSO) machine learning method with high accuracy, which will be helpful to future researchers in predicting biomass and leaf area with high accuracy and robustness. We used the broad genotypic base to include all possible variations in the biomass estimation model to make it more robust. In order to bring out novelty in our research, we used 16 machine-learning models to identify the best estimation model. Wide range of phenotypic variations were taken into consideration by mixing genotypes and recombinant inbred lines (RILs) at different phenological stages. We predicted SA with higher accuracy in wheat crop having compact plant architecture which had not been validated earlier. We considered consecutive-year data set to evaluate the model replicability. We found Bayesian Least Absolute Shrinkage and Selection Operator (BLASSO) to be the best model which gives prediction accuracy of 0.96 for FW, 0.90 for DW and 0.96 for SA. So, this model can be used as a generic model to predict the vegetative stage biomass and leaf area in wheat. The use of leaf area for estimation of conventional physiological parameters such as leaf area index will be useful in crop improvement programmes. As unique research, it included many biologically relevant image-based traits, including NIR mean gray value, RGB colour class data and mean RGB-derived indices. This work showed the vast potential for future applicability in discovering novel QTLs for biomass and growth-related traits at different phenological stages. Precise phenotyping of biomass at multiple time points in the plant life cycle will serve as a seed for the functional mapping of dynamic traits. It also has future implications in characterizing and quantifying nutrient deficiency effects at the different phenological time scale.

## Data availability statement

The original contributions presented in the study are included in the article/**Supplementary Material**. Further inquiries can be directed to the corresponding author.

## Author contributions

The manuscript was reviewed and approved for publication by all authors. VC, SK and BS: Conceptualization and designing of experiment. BS: Final data analysis, interpretation and writing-original draft. BS, SK, DV, SA, PS and GP: Experiment setup, data collection and data input. BS, AE and ND: Formal data analysis and visualization. VC, SK, HK, RS, DR, MD and LS: Performance evaluation and review of whole experiment. VC, SK, HK, RS, DR, MD and LS: Revision of manuscript. All authors contributed to the article and approved the submitted version.
