# Supplementary material for: Phenomics based prediction of plant biomass and leaf area in wheat using machine learning approaches
Source: Front Plant Sci. 2023 Jun 28;14:1214801. doi: 10.3389/fpls.2023.1214801 (PMC10337996; doi:10.3389/fpls.2023.1214801)
Supplement: Supplementary file 1 [file DataSheet_1.pdf]

Model Details

ML Code	R package	Abbreviation
BGLM	arm	Bayesian Generalized Linear Model
BLASSO	monomvn	Bayesian Lasso
BRNN	brnn	Bayesian Regularized Neural Networks
GBM	gbm, plyr	Stochastic Gradient Boosting
GLM		Generalized Linear Model
GLMNET	glmnet, Matrix	Lasso and Elastic-Net Regularized Generalized Linear Models
GP-Poly	kernlab	Gaussian Process with Polynomial Kernel
GP-Radial	kernlab	Gaussian Process with Radial Kernel
KNN	kknn	k-Nearest Neighbors
LASSO	elasticnet	Lasso Model
MARS	earth	Multivariate Adaptive Regression Spline
MLR		Multivariate Linear Regression
RF	randomForest	Random Forest
RIDGE	elasticnet	Ridge Regression
SVM-Linear	e1071	Support Vector Machines with Linear Kernel
SVM-Radial	kernlab	Support Vector Machines with Radial Kernel

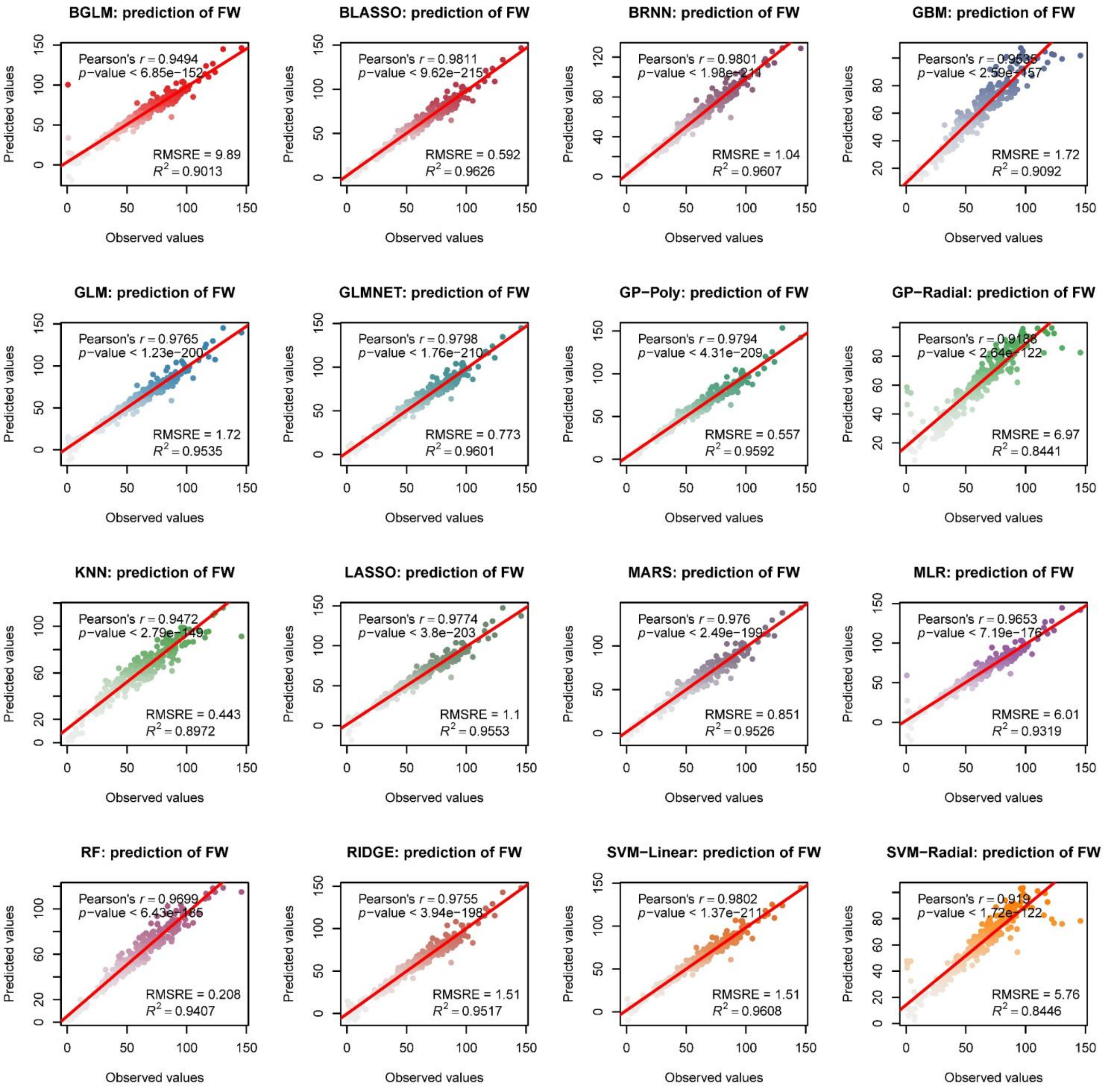

Figure: Performance of all 16 machine leaning models for prediction of fresh weight in experiment 1.

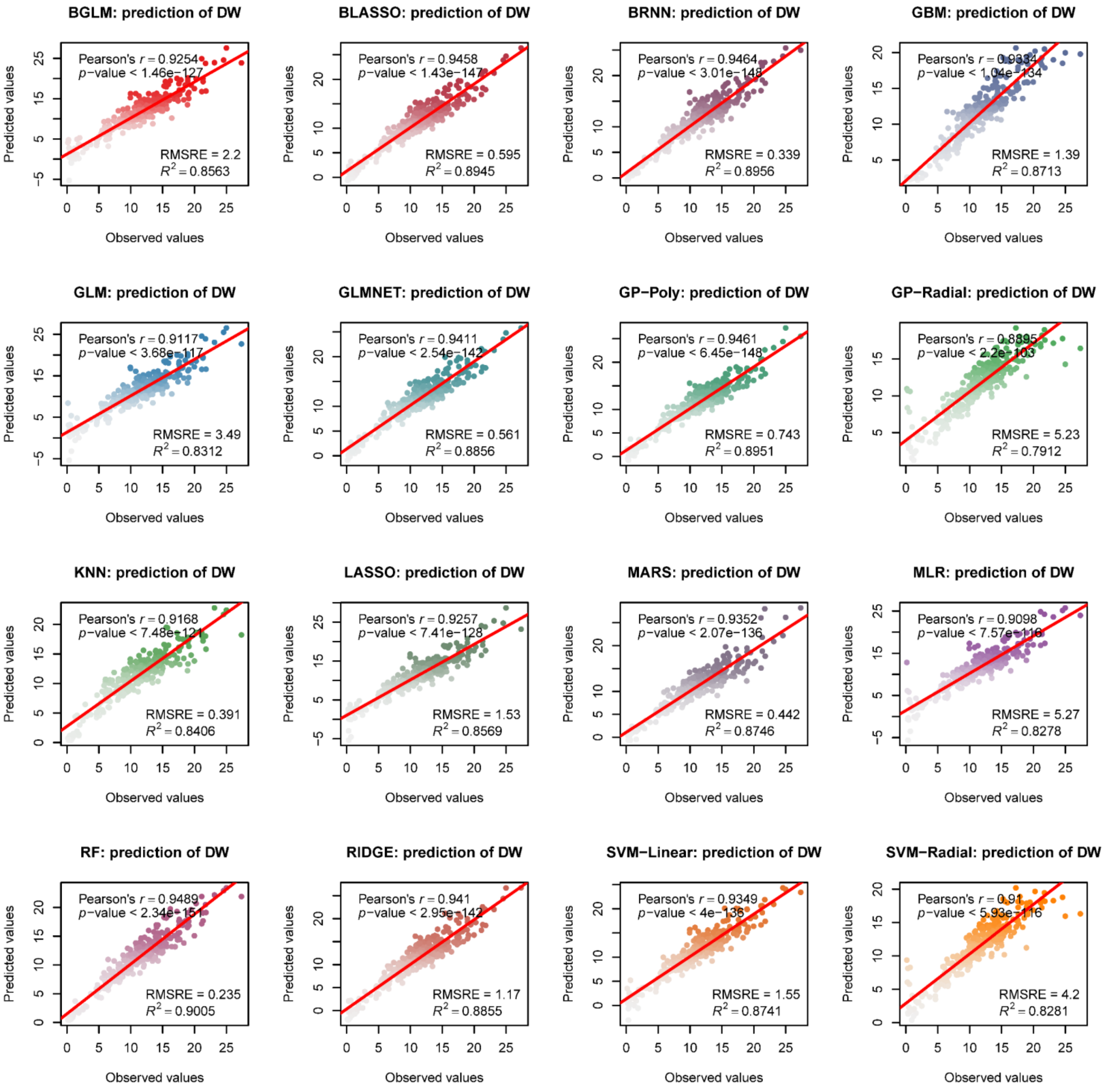

Figure: Performance of all 16 machine leaning models for prediction of dry weight in experiment 1.

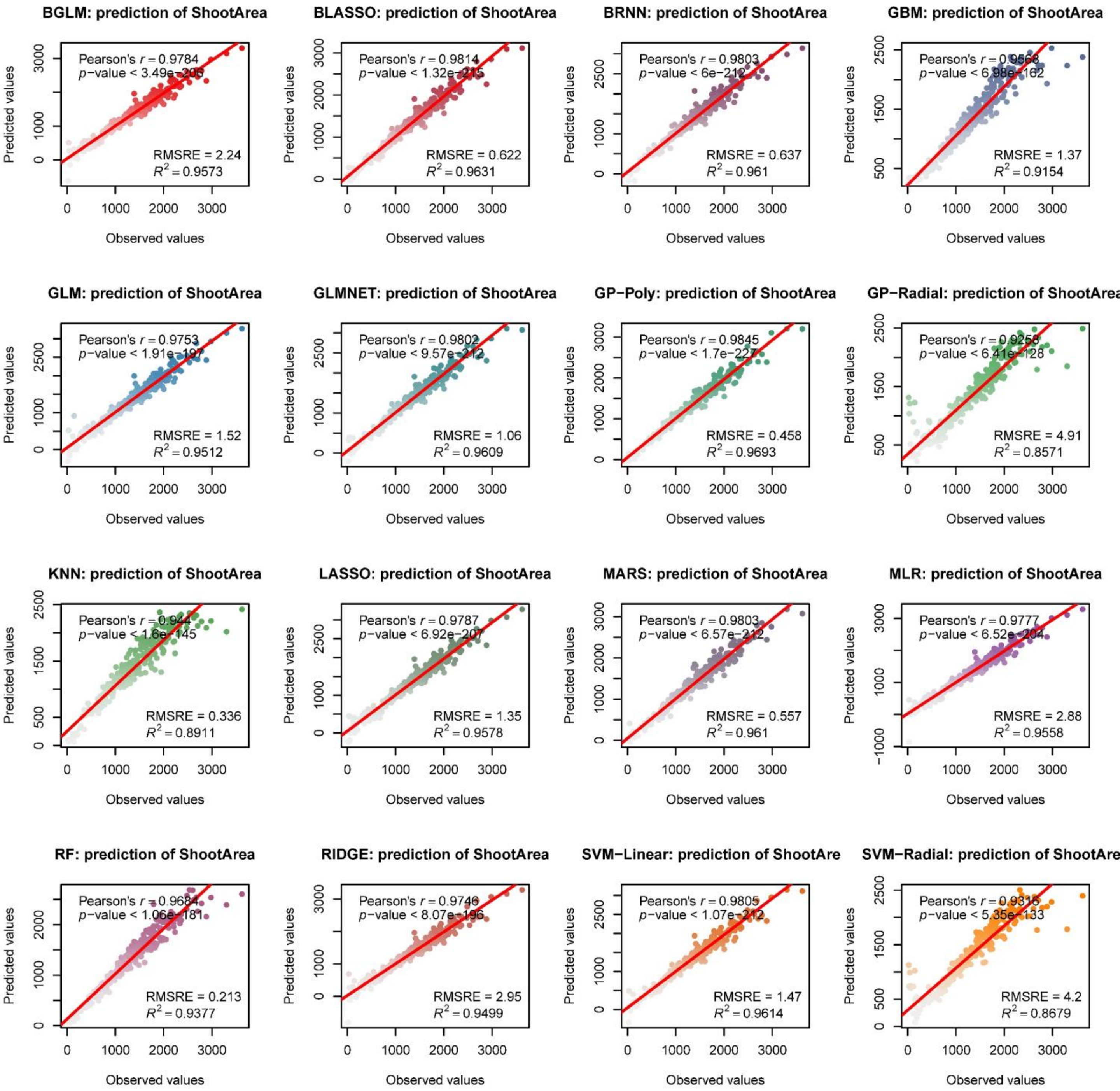

Figure: Performance of all 16 machine leaning models for prediction of shoot area in experiment 1.

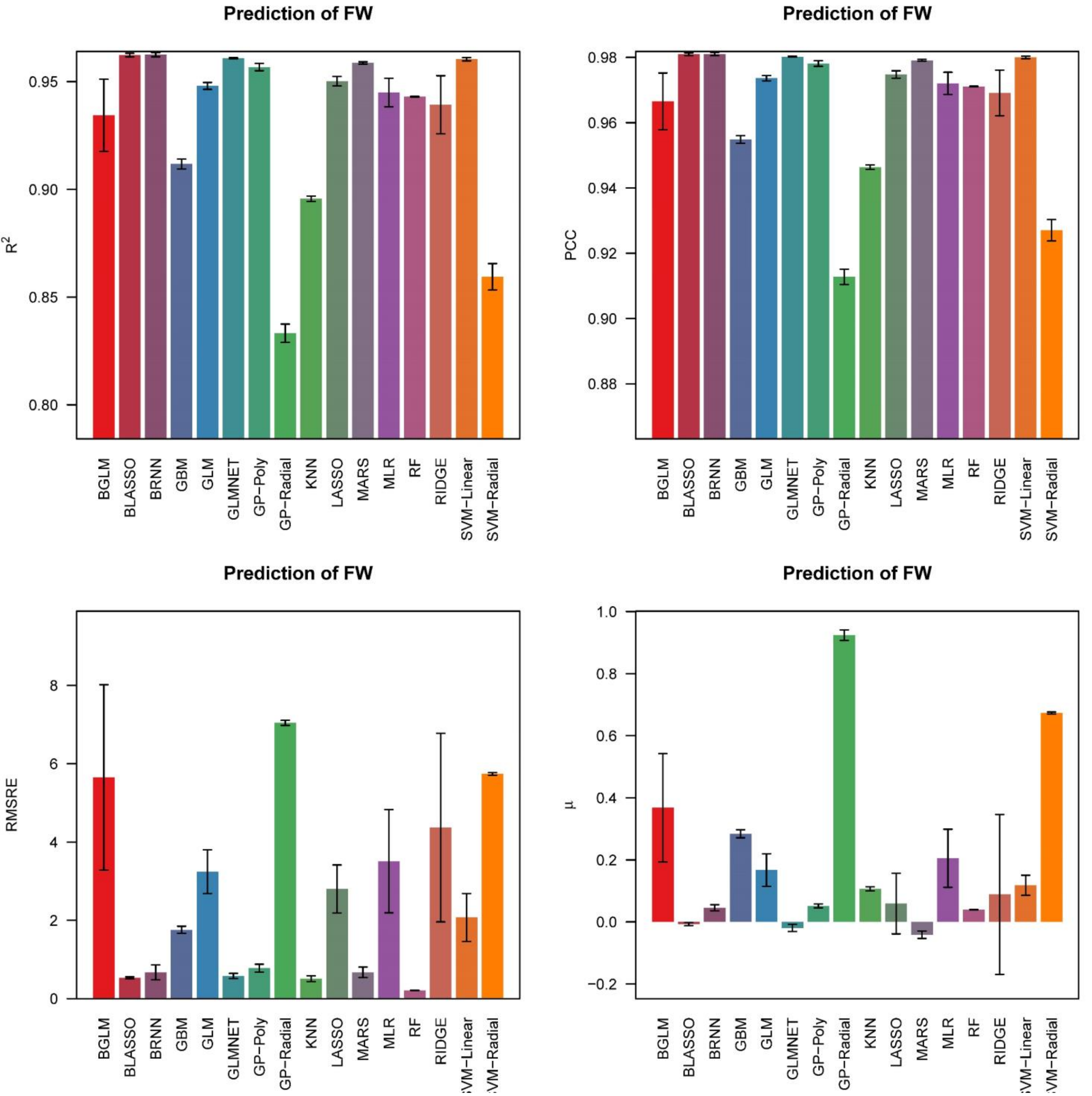

Figure: Performance evaluation of all 16 machine leaning models for prediction of Fresh weight in experiment 1.

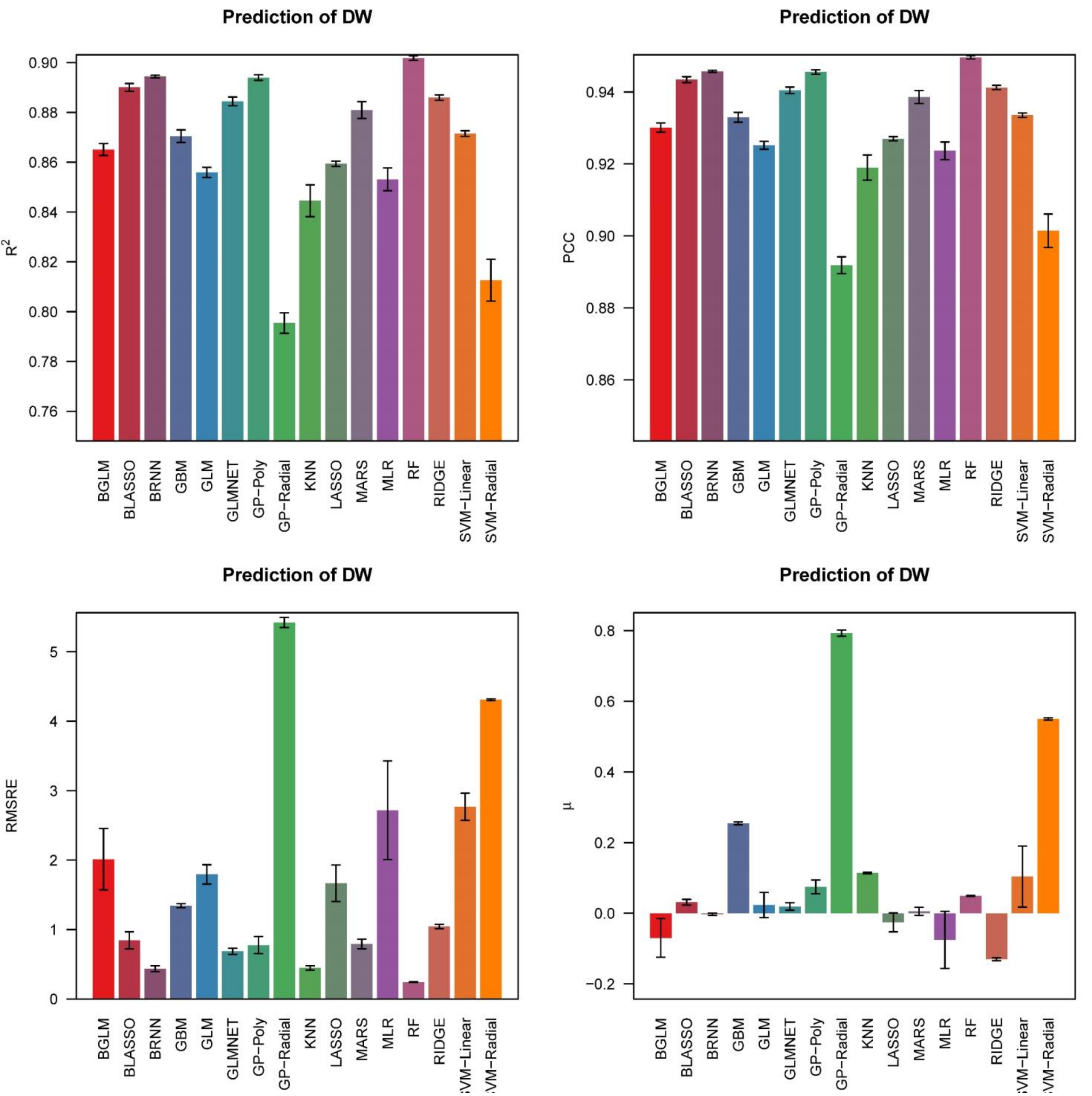

Figure: Performance evaluation of all 16 machine leaning models for prediction of Dry weight in experiment 1.

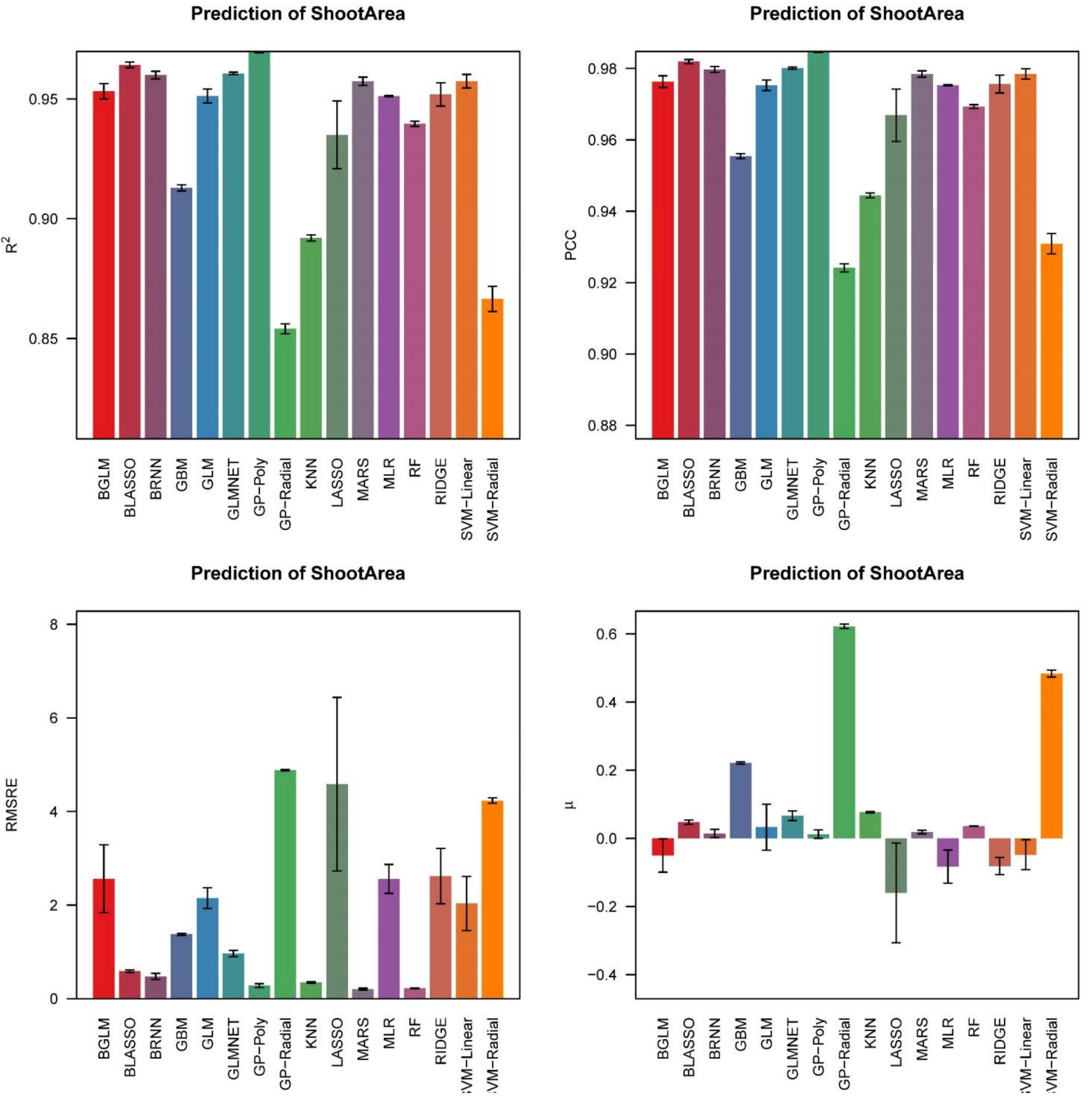

Figure: Performance evaluation of all 16 machine leaning models for prediction of Shoot area in experiment 1.

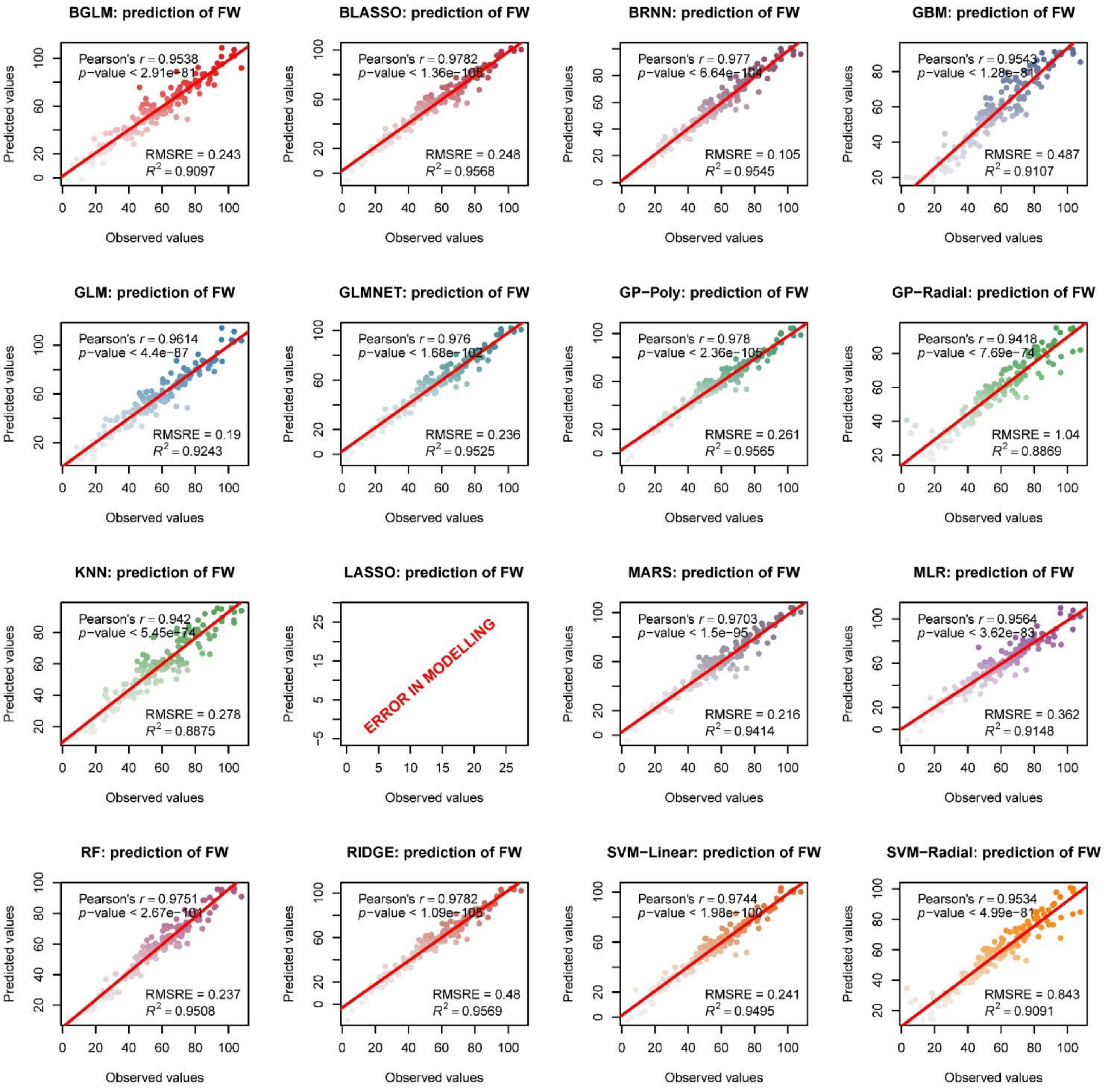

Figure: Performance of all 16 machine leaning models for prediction of fresh weight in experiment 2.

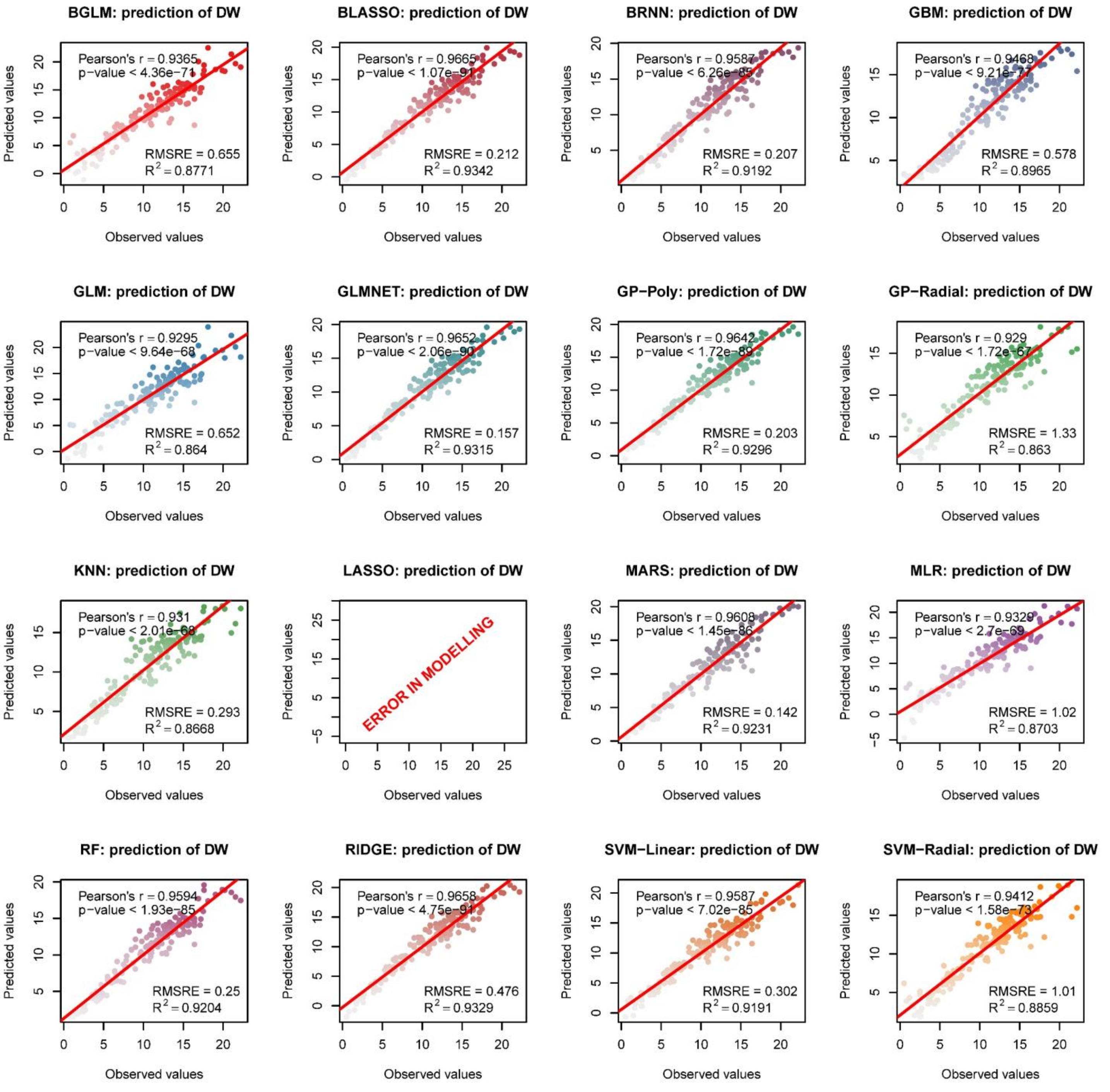

Figure: Performance of all 16 machine leaning models for prediction of dry weight in experiment 2.

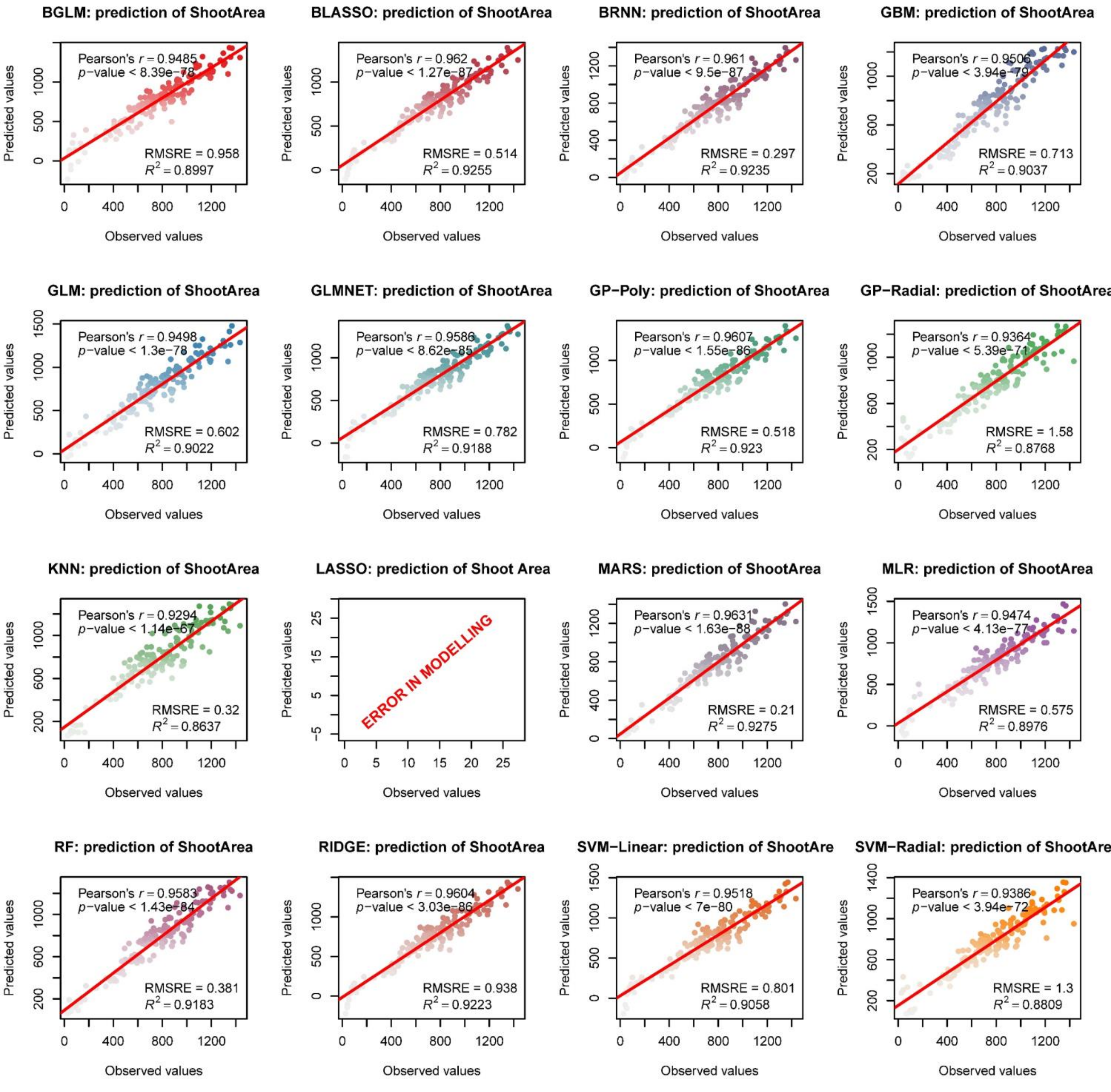

Figure: Performance of all 16 machine leaning models for prediction of shoot area in experiment 2.

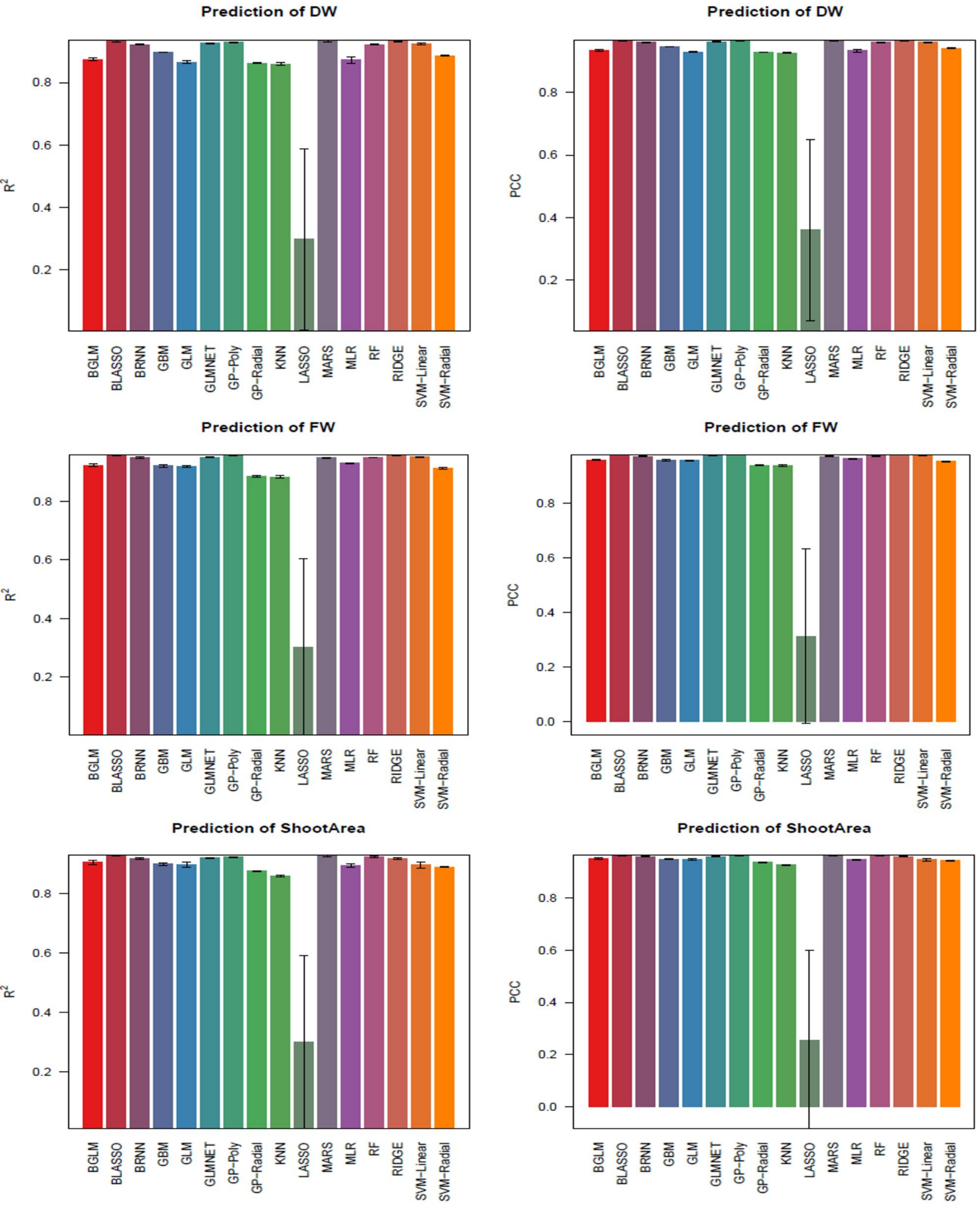

Figure: Performance evaluation of all 16 machine leaning models for prediction of FW, DW and shoot area in experiment 2.

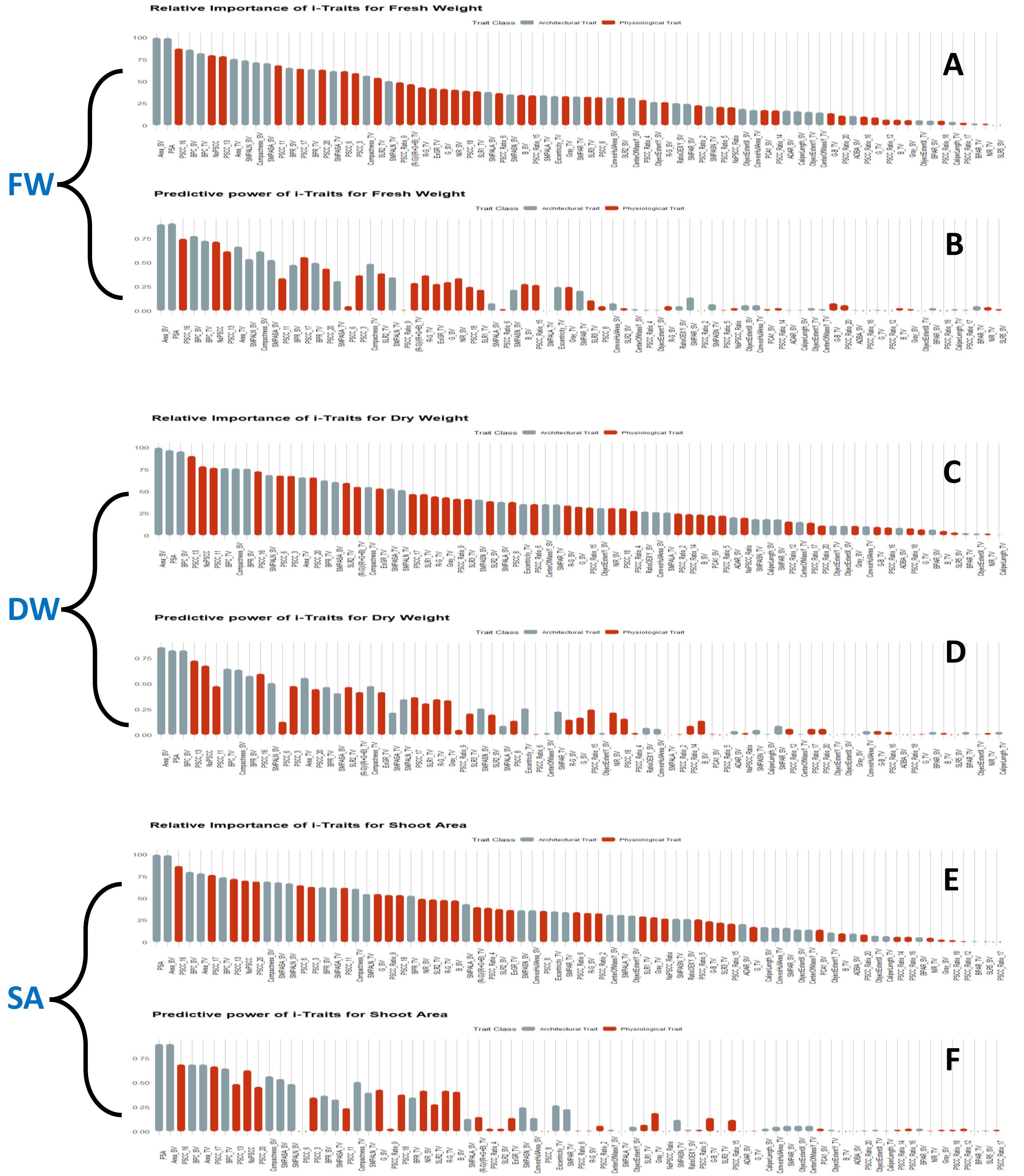

Figure: Estimation of predictive power and relative importance of i-Traits used in BLASSO model (Experiment 2). A, C and E) Relative importance of i-Traits used for FW, DW and shoot area prediction respectively. B, D and F) Predictive power of i-Traits used for FW, DW and shoot area prediction respectively.
